# Low shifts in salinity determined assembly processes and network stability of microeukaryotic plankton communities in a subtropical urban reservoir

**DOI:** 10.1186/s40168-021-01079-w

**Published:** 2021-06-03

**Authors:** Yuanyuan Mo, Feng Peng, Xiaofei Gao, Peng Xiao, Ramiro Logares, Erik Jeppesen, Kexin Ren, Yuanyuan Xue, Jun Yang

**Affiliations:** 1 Aquatic Ecohealth Group, Fujian Key Laboratory of Watershed Ecology, Key Laboratory of Urban Environment and Health, Institute of Urban Environment, Chinese Academy of Sciences, Xiamen, 361021 China; 2 University of Chinese Academy of Sciences, Beijing, 100049 China; 3 Institute of Marine Sciences, CSIC, Passeig Marítim de la Barceloneta 37-49, ES08003 Barcelona, Spain; 4 Department of Bioscience, Aarhus University, 8600 Silkeborg, Denmark; 5 Sino-Danish Centre for Education and Research, Beijing, 100049 China; 6 Limnology Laboratory, Department of Biological Sciences and Centre for Ecosystem Research and Implementation, Middle East Technical University, 06800 Ankara, Turkey; 7 Institute of Marine Sciences, Middle East Technical University, 33731 Erdemli-Mersin, Turkey

**Keywords:** Subtropical reservoir, Microeukaryotic plankton, Community ecology, Network stability, Core taxa, Satellite taxa, Salinity, Deterministic processes, Stochastic processes

## Abstract

**Background:**

Freshwater salinization may result in significant changes of microbial community composition and diversity, with implications for ecosystem processes and function. Earlier research has revealed the importance of large shifts in salinity on microbial physiology and ecology, whereas studies on the effects of smaller or narrower shifts in salinity on the microeukaryotic community in inland waters are scarce. Our aim was to unveil community assembly mechanisms and the stability of microeukaryotic plankton networks at low shifts in salinity.

**Results:**

Here, we analyzed a high-resolution time series of plankton data from an urban reservoir in subtropical China over 13 consecutive months following one periodic salinity change ranging from 0 to 6.1‰. We found that (1) salinity increase altered the community composition and led to a significant decrease of plankton diversity, (2) salinity change influenced microeukaryotic plankton community assembly primarily by regulating the deterministic-stochastic balance, with deterministic processes becoming more important with increased salinity, and (3) core plankton subnetwork robustness was higher at low-salinity levels, while the satellite subnetworks had greater robustness at the medium-/high-salinity levels. Our results suggest that the influence of salinity, rather than successional time, is an important driving force for shaping microeukaryotic plankton community dynamics.

**Conclusions:**

Our findings demonstrate that at low salinities, even small increases in salinity are sufficient to exert a selective pressure to reduce the microeukaryotic plankton diversity and alter community assembly mechanism and network stability. Our results provide new insights into plankton ecology of inland urban waters and the impacts of salinity change in the assembly of microbiotas and network architecture.

**Video abstract**

**Supplementary Information:**

The online version contains supplementary material available at 10.1186/s40168-021-01079-w.

## Introduction

Freshwater salinization (i.e., increasing salt concentration) is becoming an extensive global environmental problem potentially caused by saltwater intrusion, urbanization, and climate change, especially in semi-arid and arid climate zones [1–5]. Saltwater intrusion can jeopardize drinking water resources and infrastructure such as tubing systems, which may suffer from greater wear [6]. Severe salinization has also detrimental influences on freshwater ecosystems [7], such as lethal effects (loss of species diversity) and fitness reduction of freshwater organisms (sublethal effects) in high-salinity conditions. For example, suppression of growth [8], decreased feeding efficiency [9], and increased deformities in frogs [10] have been observed with increasing salinity. Similarly, aquatic micro-organisms tend to be negatively impacted by high salinity in hyper-saline lakes, as reflected by decreased microbial diversity [11].

Microeukaryotic plankton communities are key components of aquatic ecosystems and play a significant ecological role in controlling food web structuring and carbon flow through photosynthesis [12, 13]. Understanding the ecological processes determining the community assembly of these microorganisms is central to the field of community ecology [14, 15]. Deterministic and stochastic processes explain the assembly of microbial communities [15, 16]. Deterministic processes involve both biotic and abiotic factors, namely interspecies interactions (e.g., competition, predation, mutualism, and tradeoff) and environmental filtering (e.g., salinity, pH, temperature), which together shape community composition [17, 18]. Stochastic processes consider that all species are ecologically equivalent, and include random birth, death, dispersal, extinction, and speciation, which also affect community assembly [19, 20]. Microeukaryotic community assembly may be strongly affected by stochastic processes in rivers and oceans [21, 22]. However, there is evidence that deterministic processes may be the dominant ecological mechanisms determining the community assembly of microeukaryotic plankton at determined spatial scales [23]. In most cases, both deterministic and stochastic processes can jointly shape microeukaryotic plankton communities [24, 25]. Previous studies have revealed that broad salinity change is one of the most significant environmental variables shaping microbial community structure in aquatic and terrestrial ecosystems (see Additional file 1: Table S1). Yet, few studies have investigated the influences of low shifts in salinity on the community assembly of microeukaryotic plankton in inland freshwaters.

How environmental changes, such as salinity and drought disturbance, affect microbial assembly and the balance between deterministic and stochastic processes remains unclear. As both processes are already governing the microeukaryotic plankton community assembly in natural ecosystems, they will likely be promoted or limited by environmental changes across time and space. A previous study found that fungal community stochasticity did not increase when drought stress was relieved and attributed this to strong deterministic selection imposed by the host in the sorghum system [26]. Another study revealed that a strong selection pressure was imposed by salinity on the soil microbial community in desert ecosystems, resulting in dominance of deterministic processes under high-salinity conditions [27]. Importantly, salinity-driven selection is regarded as a major factor affecting the balance of assembly mechanisms in soil bacterial communities [28]. Evidently, temporal dynamics are often associated with changes in environmental conditions, which complicate our understanding of the mechanisms underpinning community assembly [29]. However, few studies have explored the change in deterministic processes relative to the changes in stochastic counterparts induced by low shifts in salinity in inland freshwaters.

Further, environmental disturbances likely destabilize microbial co-occurrence networks [30]. In natural ecosystems, most microeukaryotic plankton communities consist of a few core taxa with high abundances and a huge number of satellite taxa with low abundances [31, 32]. A previous study hypothesized that in macro-organisms core taxa are mainly influenced by selection, whereas satellite taxa are mostly affected by dispersal limitation [33]. Such partition has been valuable for comprehending the ecological processes shaping macro-organism communities [34, 35], thereby contributing to a better understanding of ecosystem functions [36]. There is increasing evidence indicating that community responses to environmental disturbances can be affected by ecological network characteristics [37]. For example, weak interactions and co-exclusion lead to more stable temporal ecological networks [38]. Despite a surge of new and insightful network analyses in ecology, significant knowledge gaps remain regarding how microeukaryotic plankton community stability responds to low shifts in salinity.

**Fig. 1:**
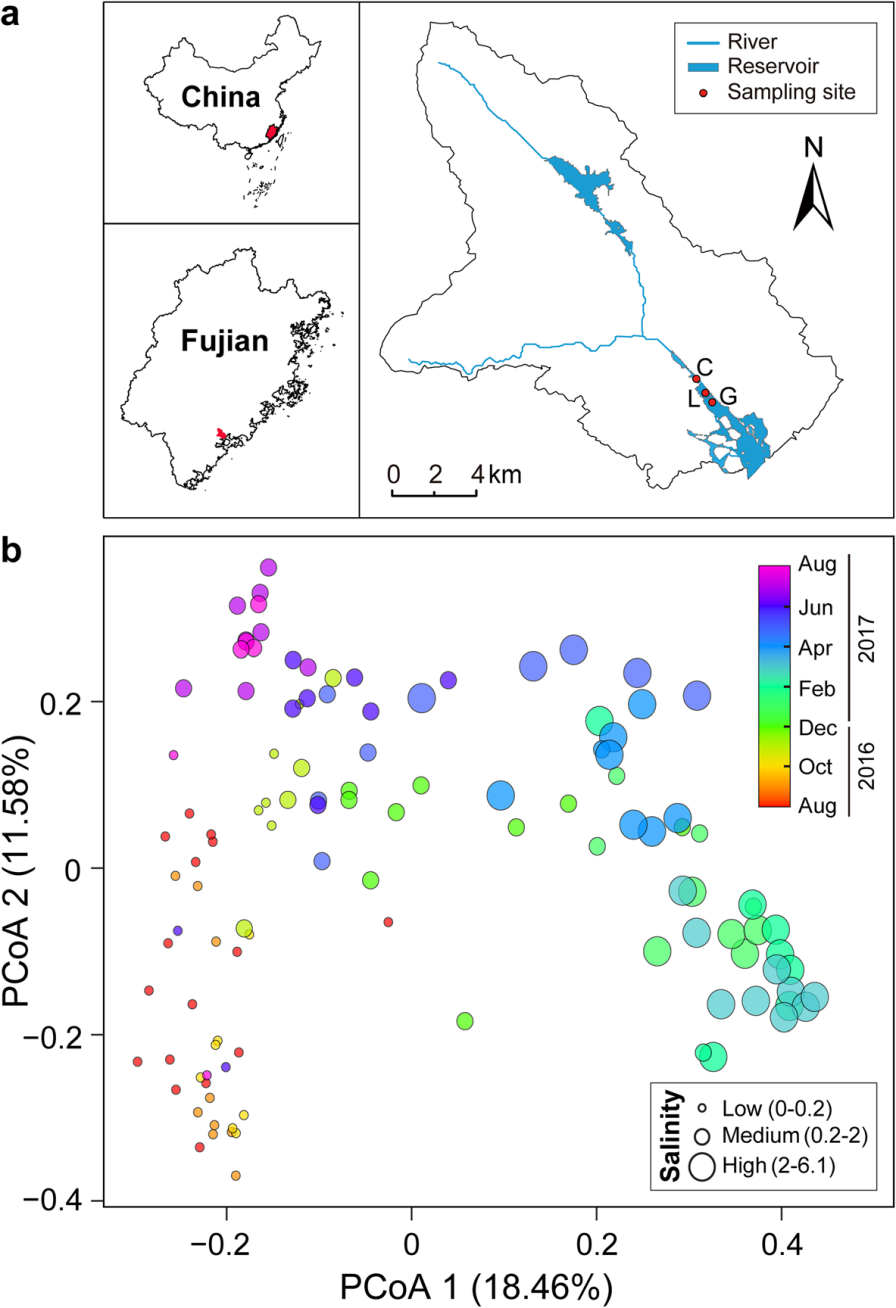
Sampling sites and principal coordinates analysis of the microeukaryotic plankton community. **a** Location of the three sampling sites in Xinglinwan Reservoir, Xiamen City, Southeast China. Water samples were taken from stations C, L, and G. **b** Principal coordinates analysis (PCoA) of microeukaryotic plankton community composition at station G in the Xinglinwan Reservoir. Each circle represents one sample and is color-coded according to time (month) and sized according to salinity

Estuarine or brackish waters are ideal systems for elucidating microbial dynamics with changing salinity [14, 39, 40]. However, unlike estuaries and saline lakes, our study area, the Xinglinwan Reservoir, is a freshwater urban reservoir in a rapidly urbanizing area (Jimei District of Xiamen City) of southeast China, subjected to periodic salinization. The resident population in Jimei district of Xiamen City jumped from 148,000 in 2000 to about 580,000 in 2010, and then to 1037,000 in 2020. After the construction of the Xingji seawall in 1979, the Xinglinwan Reservoir gradually evolved from the natural bay to the present enclosed water body [41]. The reservoir was disconnected from the ocean by a sluicegate [41], which may be the main factor causing the salinity changes observed in this water body. In addition, terrestrial input, water discharge from surrounding human activities, and precipitation may also have contributed to salinity changes [42]. The Xinglinwan Reservoir is the most important water body in the Jimei District and plays an important role for the landscape, water storage, and flood control in Xiamen City. Yet, it is not clear how low shifts in salinity in the reservoir affect the diversity and community assembly of microeukaryotic plankton as well as their co-occurrence patterns. Here, we examined the dynamics of microeukaryotic plankton communities in this subtropical urban reservoir using 18S rRNA gene sequencing based on high-frequency sampling (daily to weekly) over a 13-month period. To facilitate comparison between different salinity levels at low shifts in salinity, we artificially divided the samples into three salinity categories: low-salinity (0–0.2‰), medium-salinity (0.2–2‰), and high-salinity (2–6.1‰) conditions (Fig. 1a, b).

Two hypotheses were tested: (i) low shifts in salinity at low salinities would significantly affect the composition and diversity of the microeukaryotic plankton communities in freshwaters through a progressive increase in deterministic processes and a decrease in stochastic counterparts; (ii) the co-occurrence network stability of core and satellite plankton subcommunities would be different with low shifts in salinity.

## Results

### Temporal dynamics of environmental variables

A suite of environmental factors characterizing the Xinglinwan Reservoir are visualized in Figures S1 and S2 in Additional file 1. Compared with station C, several similar temporal tendencies of the physical and chemical factors were found at stations L and G. Salinity, electrical conductivity (EC), and total nitrogen (TN) showed almost synchronous changes over time, while salinity and precipitation/temperature demonstrated the opposite trend (Additional file 1: Figure S2). Furthermore, opposite trends were observed for salinity and precipitation between 2016 and 2018, and Spearman’s correlation indicated that salinity and precipitation exhibited a significant and negative correlation from 2016 to 2018 (Additional file 1: Figure S3).

### Composition and temporal dynamics of microeukaryotic plankton communities

The total number of microeukaryotic OTUs was 19,952 in the reservoir at 97% similarity level, which included 618 core OTUs and 18,056 satellite OTUs (Additional file 1: Table S2, Additional file 1: Figure S4a, b). Continuous fluctuations were observed in each plankton supergroup of the microeukaryotic community, and the most abundant OTUs were assigned to the groups of Alveolata, Archaeplastida, Cryptista, Opisthokonta, and Stramenopiles (Additional file 1: Figure S5a). In addition, the core taxa were more abundant but less diverse than the satellite taxa at phylum level (Additional file 1: Figure S5b). The absolute abundance of the microeukaryotic communities ranged from 4.19 × 10^9^ to 4.04 × 10^10^ copies/L at station C, while the absolute abundance at stations L and G varied from 4.21 × 10^9^ to 7.92 × 10^10^ copies/L and from 4.29 × 10^8^ to 6.63 × 10^11^ copies/L, respectively. The fluctuations in absolute abundance of microeukaryotes in the time series were larger than that of bacterioplankton (Additional file 1: Figure S6a, b). Changes in the ratio of microeukaryotic 18S rRNA gene to bacterial 16S rRNA gene were observed across the time series (Additional file 1: Figure S6c), however the abundance of microeukaryotic 18S rRNA gene exhibited a significant positive correlation with bacterial 16S rRNA gene at stations C, L, and G, respectively (Additional file 1: Figure S6d).

Non-metric multi-dimensional scaling (NMDS) ordination showed a significant segregation of whole-community microeukaryotic OTUs (based on 97% sequence similarity) between the three salinity levels at stations C, L, and G, whereas the microeukaryotic communities at stations C, L, and G exhibited a significant aggregation at low-salinity conditions, indicating no significant spatial difference in the effects of salinity on community composition. Whole-community ASVs (amplicon sequence variants) results showed almost identical pattern as those based on OTUs (ρ = 0.997, *P* = 0.001, Additional file 1: Figure S7). Therefore, we selected only one station, station G, for high-frequency monitoring during 13 consecutive months to explore the shifts of microeukaryotic OTUs (Additional file 1: Figure S7).

### The importance of salinity in structuring plankton communities

**Fig. 2:**
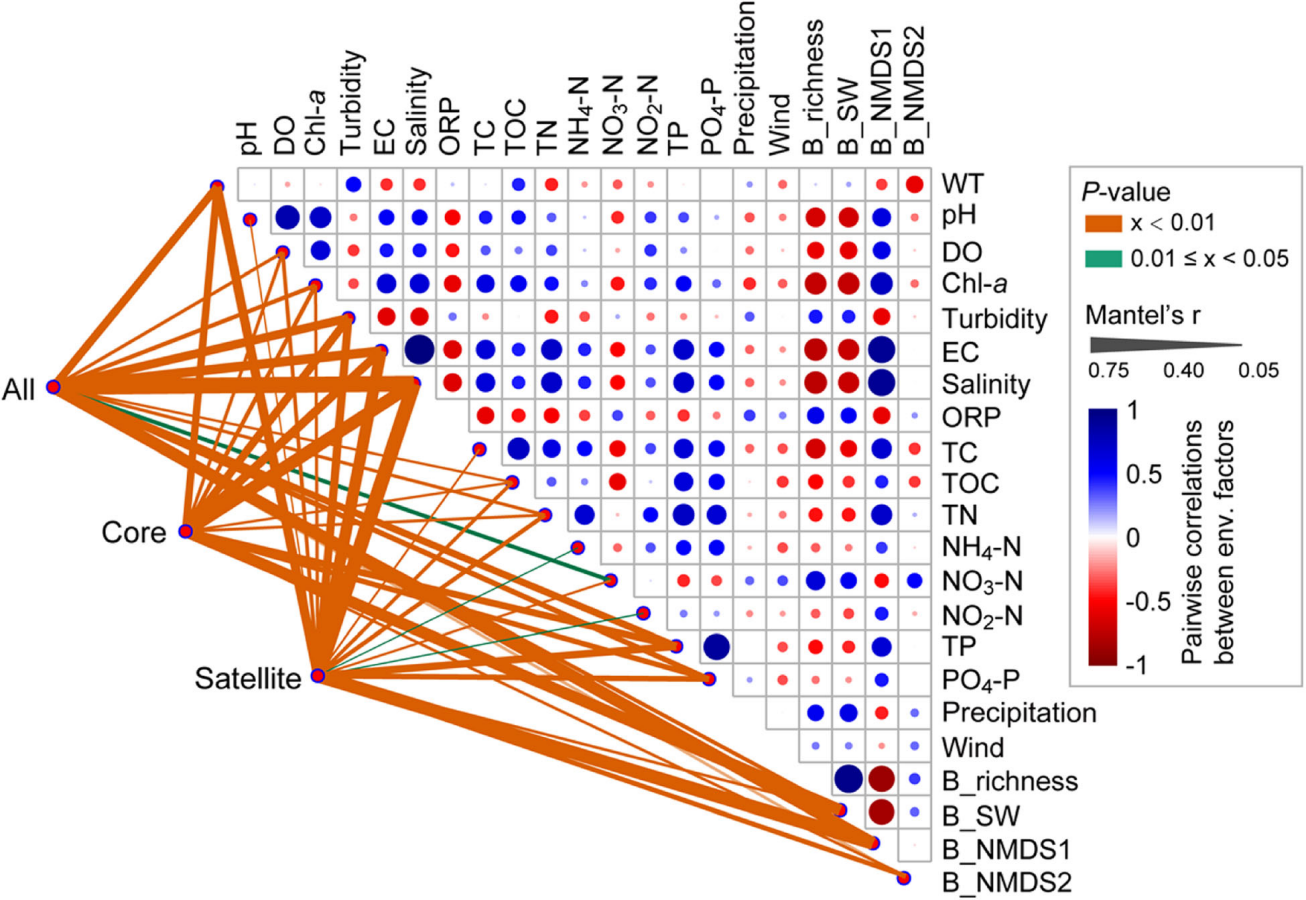
Abiotic and biotic drivers of microeukaryotic plankton community composition. Pairwise comparisons of environmental and biotic factors are shown at the upper-right, with a color gradient representing Spearman’s correlation coefficients. Microeukaryotic plankton community composition was correlated to each environmental or biotic factor by partial Mantel tests. The line width represents the partial Mantel’s r statistic for the corresponding correlation, and line color means that significances are tested based on 999 permutations. WT, water temperature; DO, dissolved oxygen; Chl-*a*, chlorophyll-*a*; EC, electrical conductivity; ORP, oxidation-reduction potential; TC, total carbon; TOC, total organic carbon; TN, total nitrogen; NH_4_-N, ammonium nitrogen; NO_3_-N, nitrate nitrogen; NO_2_-N, nitrite nitrogen; TP, total phosphorus; PO_4_-P, phosphate phosphorus; Note that the precipitation data are the 7-day accumulation before the sampling day, and the wind represents daily average wind speed. B_richness, bacterial OTU number; B_SW, bacterial Shannon-Wiener index; B_NMDS1, bacterial NMDS ordination axis 1; B_NMDS2, bacterial NMDS ordination axis 2. Note that only significant correlations are shown for simplicity

Microeukaryotic plankton communities were separated based on salinity or time (month) at station G (Fig. 1). Our results showed that salinity exhibited the strongest correlations with all, core, and satellite microeukaryotic community composition, followed by alpha- and beta-diversities of bacterioplankton (Fig. 2). Other significant environmental variables were water temperature, pH, dissolved oxygen, chlorophyll-*a*, turbidity, electrical conductivity, total carbon, total organic carbon, total nitrogen, ammonium nitrogen, nitrate nitrogen, nitrite nitrogen, total phosphorus, and phosphate phosphorus (Fig. 2). In addition, the all community Bray-Curtis dissimilarity exhibited a stronger correlation with salinity (*R* = 0.774, *P* < 0.01) than with time (*R* = 0.658, *P* < 0.01 for absolute time; *R* = 0.646, *P* < 0.01 for annual cycle time) (Additional file 1: Figure S8). The Mantel and partial Mantel results also revealed that both salinity and time significantly explained the change in microeukaryotic community composition (including all, core, and satellite taxa; *P* < 0.01), whereas salinity had a greater influence on microeukaryotic communities than time (Additional file 1: Table S3). Furthermore, the effects of salinity on alpha-diversity of all, core, and satellite plankton were stronger than time or the interaction of salinity and time (Table 1).

**Table 1:** Two-way ANOVA showing the effects of time and salinity on the alpha-diversity of microeukaryotic plankton communities

	All		Core		Satellite	
	F	*P*	F	*P*	F	*P*
Time (*n* = 13)						
Richness	2.978	**0.001**	1.957	**0.037**	3.337	**0.000**
ACE	3.201	**0.001**	1.654	0.090	3.555	**0.000**
Chao 1	3.152	**0.001**	1.622	0.098	3.479	**0.000**
Shannon-Wiener	2.431	**0.008**	1.990	**0.033**	0.931	0.520
Simpson	0.870	0.580	0.246	0.995	0.564	0.866
Pielou’s evenness	2.086	**0.025**	4.640	**0.000**	0.445	0.941
Salinity (*n* = 3)						
Richness	16.767	**0.000**	20.729	**0.000**	11.986	**0.000**
ACE	16.867	**0.000**	19.355	**0.000**	13.914	**0.000**
Chao 1	16.366	**0.000**	20.885	**0.000**	12.492	**0.000**
Shannon-Wiener	15.628	**0.000**	8.396	**0.000**	15.216	**0.000**
Simpson	3.429	**0.036**	0.359	0.699	0.684	0.507
Pielou’s evenness	10.873	**0.000**	14.707	**0.000**	1.615	0.204
Time × salinity						
Richness	3.991	**0.002**	2.550	**0.033**	3.084	**0.013**
ACE	3.951	**0.003**	2.194	0.061	3.456	**0.006**
Chao 1	3.869	**0.003**	2.054	0.078	3.223	**0.010**
Shannon-Wiener	4.528	**0.001**	2.379	**0.044**	2.294	0.051
Simpson	0.773	0.571	0.343	0.885	0.916	0.474
Pielou’s evenness	3.676	**0.004**	4.691	**0.001**	0.487	0.785

Boldface means significance at *P* < 0.05 level

Time includes 13 successional months

Salinity includes low, medium, and high salinity levels

*All* all microeukaryotic plankton communities, *Core* core microeukaryotic plankton subcommunities, *Satellite* satellite microeukaryotic plankton subcommunities

### Microeukaryotic plankton community composition and diversity along salinity gradient

**Fig. 3:**
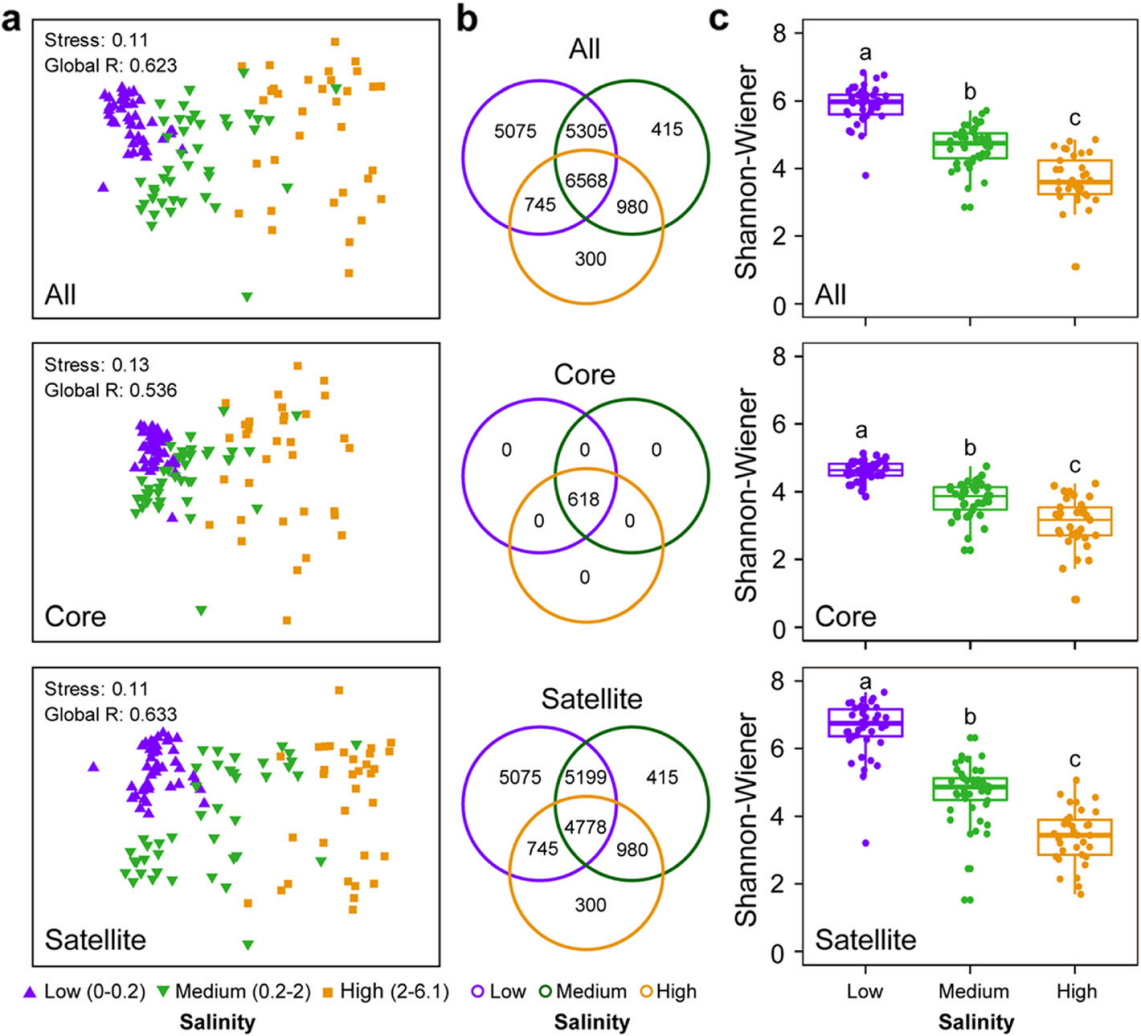
Community structuring of microeukaryotic plankton across the salinity gradient at station G in Xinglinwan Reservoir. **a** Non-metric multidimensional scaling (NMDS) ordination based on Bray-Curtis dissimilarity showing the variation of microeukaryotic plankton communities across three salinity levels. Significant level of all, core, and satellite taxa is *P* = 0.001. **b** Venn diagram showing the numbers of unique and shared OTUs between three different salinity levels. **c** Shannon-Wiener index along the salinity level at station G. Different letters indicate significant difference at *P* < 0.05 according to Tukey’s post-hoc test. All, all microeukaryotic plankton communities at station G; Core, core microeukaryotic plankton subcommunities at station G; Satellite, satellite microeukaryotic plankton subcommunities at station G

Non-metric multi-dimensional scaling (NMDS) ordination and ANOSIM tests showed that the composition of the microeukaryotic plankton communities (all, core, and satellite taxa) differed significantly between low-, medium-, and high-salinity levels (*R* = 0.623, *P* = 0.001 for all taxa; *R* = 0.536, *P* = 0.001 for core taxa; and *R* = 0.633, *P* = 0.001 for satellite taxa, Fig. 3a). Further, all core OTUs were shared among the three salinity levels, while the proportion of shared OTUs (27.3%) was much lower for the satellite taxa (Fig. 3b). Shannon-Wiener diversity decreased with increasing salinity for all, core, and satellite plankton taxa (Fig. 3c), with Chlorophyta and Ochrophyta being the dominant groups. The relative abundance of Chlorophyta and Ochrophyta increased and decreased, respectively, with increasing salinity, especially for all and core taxa (Additional file 1: Figure S9).

### Relative importance of deterministic and stochastic processes along salinity gradient

**Fig. 4:**
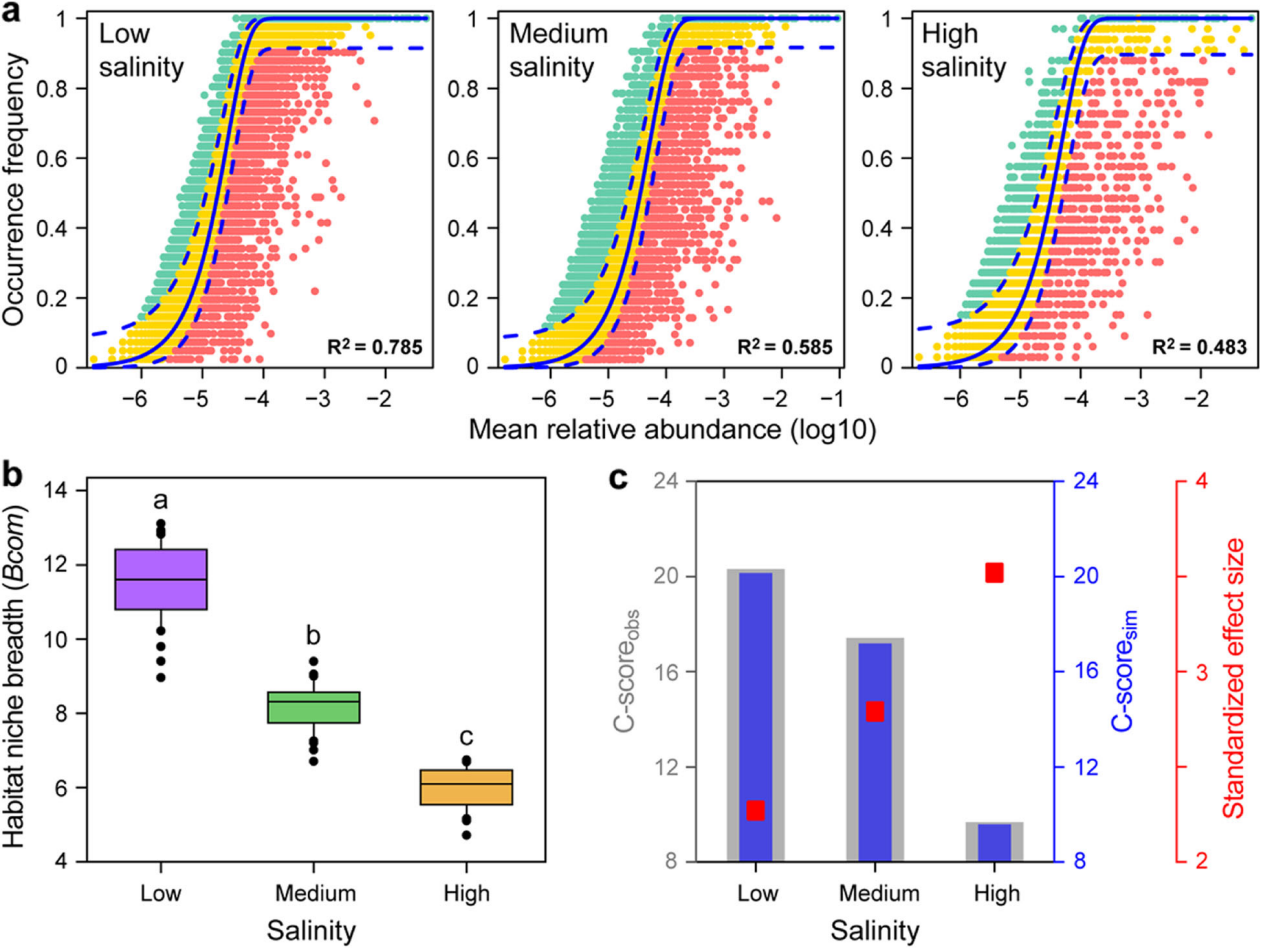
Ecological processes shaping the microeukaryotic plankton community assembly at station G in Xinglinwan Reservoir. **a** The predicted occurrence frequencies for low, medium, and high salinity representing microeukaryotic plankton communities from low, medium, and high salinity periods in Xinglinwan Reservoir. The solid blue line is the best fit to the neutral community model (NCM), and the dashed blue line indicates 95% confidence intervals around the NCM prediction. OTUs that occur more or less frequently than predicted by the NCM are shown in green and red, respectively. R^2^ represents the fit to this model. **b** Comparison of mean habitat niche breadth for all taxa among low, medium, and high salinity levels (different letters indicate significant difference at the *P* < 0.05 level using Tukey’s post hoc test). **c** C-score metric using null models. The values of observed C-score (C-score_obs_) > simulated C-score (C-score_sim_) indicate non-random co-occurrence patterns. Standardized effect size <− 2 and > 2 represent aggregation and segregation, respectively

The relationship between the occurrence frequency of OTUs and their relative abundance was well described by the neutral community model (Fig. 4a). The relative contribution of stochastic processes decreased gradually with increasing salinity, explaining 78.5%, 58.5%, and 48.3% of the community variance for the low, medium, and high-salinity levels, respectively. The same pattern was observed in the succession time series, reflecting that the contribution of stochastic processes to the plankton community was low when salinity was high (Additional file 1: Figure S10). Further, all microeukaryotic plankton communities exhibited significantly wider niche breadths at low salinity than at medium-/high-salinity levels (Fig. 4b). The average niche breadth was significantly higher for core than for the satellite subcommunities (31.5 for core taxa, 10.5 for satellite taxa; *P* < 0.001) (Additional file 1: Figure S11). More importantly, the C-score showed that standardized effect size (SES) increased with increasing salinity, indicating the enhanced importance of deterministic processes for the plankton assemblage (Fig. 4c).

### Co-occurrence networks and stability of microeukaryotic plankton communities along salinity gradient

A metacommunity co-occurrence network was constructed based on all datasets from station G, and three subnetworks along three salinity levels (low, medium, and high) were analyzed for all, core, and satellite taxa (Additional file 1: Table S4), respectively. The topological properties of the networks varied significantly with salinity. For example, both nodes (i.e., OTUs) and edges (i.e., significant links or correlations between OTUs) constituting the all and core networks decreased with increasing salinity (that is, the networks became smaller with increasing salinity). Also, the satellite networks consisted of different nodes linked by different edges along salinity levels, 316 nodes being linked by 755 edges, 290 nodes by 1316 edges, and 166 nodes by 582 edges in low-, medium-, and high-salinity levels, respectively (Additional file 1: Table S4). Further, all network degrees followed a power-law distribution rather than a Poisson distribution, suggesting that the network structure exhibited a scale-free and non-random distribution (Additional file 1: Table S4). The observed network parameters (average clustering coefficient, average path length, and modularity index) were all larger than those of their respective Erdös-Réyni random networks, indicating “small-world” properties and modular structure (Additional file 1: Table S4). Based on the all network, the nodes with the top three highest degrees were OTU_3 (Fungi), OTU_37 (Chlorophyta), and OTU_98 (Ochrophyta), being potential keystone species. Both OTU_3 and OTU_37 belonged to the core microeukaryotic plankton (Additional file 1: Figure S12a). Random forest (RF) analysis indicated that the beta-diversity of satellite subcommunities exhibited a stronger relationship with the multi-nutrient cycling index than the core subcommunities (Additional file 1: Figure S12b).

**Fig. 5:**
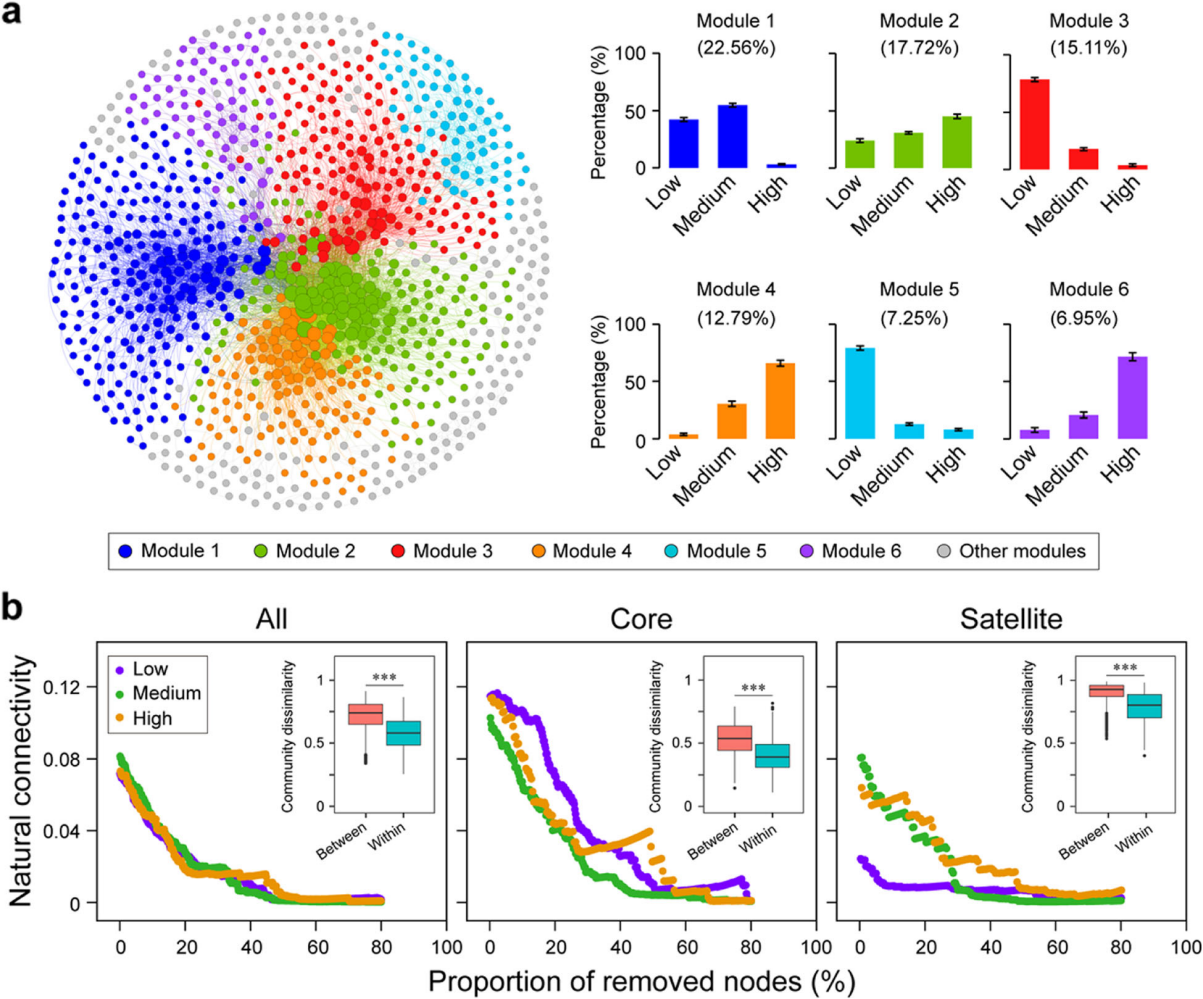
Network modules and stability for microeukaryotic plankton OTUs at station G. **a** Network revealing the modular associations among microeukaryotic plankton OTUs (left). Relative abundance of microeukaryotic OTUs from major modules along the three different salinity levels (right). A connection indicates a strong (SparCC |r| > 0.6) and significant (*P* < 0.01) correlation. The size of each microeukaryotic OTU (node) is proportional to the number of connections (i.e., degree). **b** Network stability for all, core and satellite taxa at different salinity levels (i.e., low, medium, and high), respectively. The robustness of all, core, and satellite networks under different salinity conditions in Xinglinwan Reservoir. Insert: overview of pairwise community dissimilarity of microeukaryotic plankton communities at three different salinity levels. Statistical analysis is non-parametric Mann-Whitney *U* test. ***P < 0.001. All, all microeukaryotic plankton communities; Core, core microeukaryotic plankton subcommunities; Satellite, satellite microeukaryotic plankton subcommunities

The plankton community network was clearly divided into six major modules that accounted for 82.4% of the whole network (Fig. 5a). For example, microeukaryotic plankton communities were dominated by taxa preferring medium salinity in module I, by taxa preferring high salinity in modules II, IV, and VI, and taxa preferring low salinity in modules III and V. Further, we found that the contribution of Ochrophyta, Perkinsea, and Ciliophora was higher in modules representing low salinity. However, in the modules corresponding to medium and high-salinity levels, Chlorophyta exhibited the highest degree of centrality (Additional file 1: Table S5). This indicates that Ochrophyta, Perkinsea, and Ciliophora play a key role in maintaining taxa coexistence in low-salinity network, whereas Chlorophyta are more important in maintaining coexistence in the medium and high-salinity networks because nodes with a higher degree of centrality are more important in maintaining taxa coexistence in networks.

Finally, we compared network stability between different salinity levels with varying microeukaryotic plankton subnetwork structure. Compared with core taxa, the dissimilarity of subnetworks was larger between the low and high-salinity levels for both all and satellite taxa (Additional file 1: Table S6). In fact, the community dissimilarities between the groups were always higher than those within groups for the different salinity levels, and satellite subcommunities showed a higher dissimilarity when compared against all and core taxa. When comparing the core subnetwork stability among the three salinity levels, the natural connectivity at low salinity was greater than that at medium and high-salinity levels. However, natural connectivity at low salinity was much lower than at medium or high salinity for satellite plankton subnetworks (Fig. 5b). This indicates greater core subnetwork robustness at low-salinity levels, whereas the satellite subnetwork had greater robustness at the medium and high-salinity levels.

## Discussion

### Low salinity increase triggers plankton community composition change and diversity loss

In this study, we obtained new data on microeukaryotic plankton community dynamics at low shifts in salinity in an urban freshwater habitat based on high-resolution sampling. We found that salinity variation may induce compositional changes and diversity loss in the all, core, and satellite plankton communities. Plankton Shannon-Wiener diversity decreased with increasing salinity for all, core and satellite taxa, emphasizing that microeukaryotic plankton communities were not resilient when subjected to salinity variation (Fig. 3c). Plankton sensitivity to salinity may reflect the increase in extracellular osmolarity with increasing salinity [43], meaning that microeukaryotic plankton communities that are unable to adapt to osmotic stress are likely to die or become less active, leading to a decrease in alpha-diversity of the communities. This is consistent with a former study [44], showing that mycoplankton alpha-diversity was higher during low-saline periods in coastal ecosystems. However, a whole ecosystem manipulation experiment with freshwater rock pools with three salinity levels (3, 6, and 12 ‰) did not find any negative effect on bacterial alpha-diversity of a limited salinity increase [45]. The contrasting results may be related to the different microbial groups studied (bacterial community in previous study and the microeukaryotic plankton community in this study). Low salinity increase may not be sufficient to filter out or suppress many bacterial taxa but sufficed to strongly suppress many microeukaryotic plankton species, perhaps reflecting the high abundance, fast growth rates, and rapid evolutionary adaptation by bacteria [46].

### Salinity mediates the assembly processes of microeukaryotic plankton communities

Our study indicates that low shifts in salinity in freshwaters have an important influence on the assembly of all microeukaryotic plankton communities, primarily by affecting the balance between deterministic and stochastic processes. The community variation explained by stochastic processes decreased from 78.5% at low-salinity level to 48.3% at high-salinity level (Fig. 4a). In addition, the microeukaryotic plankton communities showed wider niche breadths under low salinity than at the medium-/high-salinity conditions (Fig. 4b), implying that the community assembly was more strongly influenced by deterministic processes at high salinity, likely because deterministic processes tend to have a stronger effect on habitat specialists with a narrow niche breadth than on generalists with a wide niche breadth [23, 47]. Further, C-score results showed that the value of standardized effect size (SES) increased with increasing salinity, also indicating that the community assembly was more strongly influenced by deterministic processes with increasing salinity (Fig. 4c). There are several possible explanations. First, increased allochthony may increase stochastic processes in the wet season based on other environmental factors that do not impose strong selection [48]. Specifically, precipitation events can increase the freshwater input to the Xinglinwan Reservoir, accompanied by a decrease in salinity. Meanwhile, increased precipitation may wash the micro-organisms from surrounding environmental systems (the soil or sediment, watershed, and air) into the reservoir, leading to an enhanced immigration rate and higher diversity of microbial community, which again increases stochasticity at low salinity. This agrees with the higher microeukaryotic diversity in low-salinity periods, followed by medium and high-salinity periods (Fig. 3). Second, at low salinity, freshwater microeukaryotes may be exposed to lower physiological stress, so that they can grow and reproduce more freely, resulting in dominance of stochastic processes (e.g., birth, death, and dispersal events) in community assembly, while a strong selective pressure may be exerted on the freshwater microeukaryotic plankton when salinity increases [27]. Microbes that grow well at high salinity have developed “salt-in” and “salt-out” strategies to adjust the cytoplasm to osmotic pressure [43]. “Salt-in” often involves intake of ions (e.g., K^+^ and Cl^–^), while “salt-out” maintains a low-intracellular ion concentration through pumping out inorganic ions and accumulating compatible solutes (e.g., sucrose, glycerol, and glycin) to exclude salt from the cell and thus ensure osmotic balance [43]. This would expectedly result in a higher contribution of deterministic processes to the community assembly with increasing salinity. Third, in low-salinity ecosystems with less environmental heterogeneity or with less competitive interactions between environmental generalists, the stochastic assembly mechanism is likely to overrule deterministic processes [49]. Our results suggest that the degree to which deterministic *vs.* stochastic processes shape the microeukaryotic plankton community in this reservoir is determined more by shifts in salinity rather than by seasonality (Table 1; Additional file 1: Table S3).

### Co-occurrence network stability of core and satellite microeukaryotic plankton shaped by salinity

Although the stability of plankton communities can be used to infer ecosystem functioning [12], it is unclear how plankton co-occurrence networks in an urban reservoir respond to disturbances such as small changes in salinity. In particular, the stability of core or satellite plankton subnetworks is largely unexplored [30, 50]. Our results revealed that the six network modules corresponded well with the three salinity levels (Fig. 5a). This indicates that the modular structure or property of all microeukaryotic plankton communities was sensitive to changes in salinity. Environmental heterogeneity could induce microbial modularity [51], which helps explain why the six modules became dominant at different salinity levels. Modularity can reflect competitive/synergistic relationships and niche differentiation, yielding non-random patterns of network structure, which ultimately increases the complexity of ecological networks [52]. The network modules may act as indicators of important ecological processes following the disturbance of salinity changes.

Based on the plankton network modules, we found that core OTUs play important roles in maintaining network stability (Additional file 1: Figure S12a). Similar results have been described for microbiota in metropolitan drinking water [53], agricultural soils [54], and activated sludge ecosystems [55]. Interestingly, the contribution of the beta-diversity of the satellite subcommunity to the aquatic ecosystem multi-nutrient cycling index was greater than that of the core subcommunity (Additional file 1: Figure S12b). This suggests that satellite taxa also play key roles in maintaining ecosystem functions. Our results indicate that microbial diversity affects the multi-functionality in aquatic ecosystems.

Core plankton subnetworks were less sensitive to salinity changes than satellite taxa at low-salinity variability (Fig. 5b). At low salinity, the satellite subnetworks were less stable, while core subnetworks showed a higher stability. At higher salinity, the opposite pattern was found for the stability of core and satellite plankton subnetworks, which may be explained as follows. First, the network topological parameter ‘average clustering coefficient’ was the highest for core subnetworks and the lowest for satellite sub-networks at low salinity (Additional file 1: Table S4). This implies a higher complexity of the core subnetwork and a lower complexity of the satellite subnetwork at low salinity. High complexity networks normally tend to have greater stability due to network buffering [56], so core microeukaryotic plankton subnetworks were likely more stable and satellite subnetworks more unstable at low salinity. Second, our results indicate that core taxa had wider niche breadths than satellite taxa (Additional file 1: Figure S11), meaning that they, compared with satellite taxa, can adapt to a wide range of environmental niches [57]. Consequently, core co-occurrence subnetworks exhibited strong resistance as salinity increased, while satellite subnetworks are prone to be affected by slight disturbances of salinity. This pattern could be closely associated with plankton diversity and satellite taxa richness. In other words, salinity affects plankton diversity and satellite taxa richness and, as a consequence, network structure and connectivity. Thus, it is to be expected that core and satellite plankton exhibited similar patterns in alpha- and beta-diversities but different ecosystem stability patterns along the salinity gradient. It is, therefore, important to distinguish between core and satellite taxa of microeukaryotic plankton when assessing community stability over time and future threats to ecosystem function and services in response to environmental disturbance.

## Conclusion

**Fig. 6:**
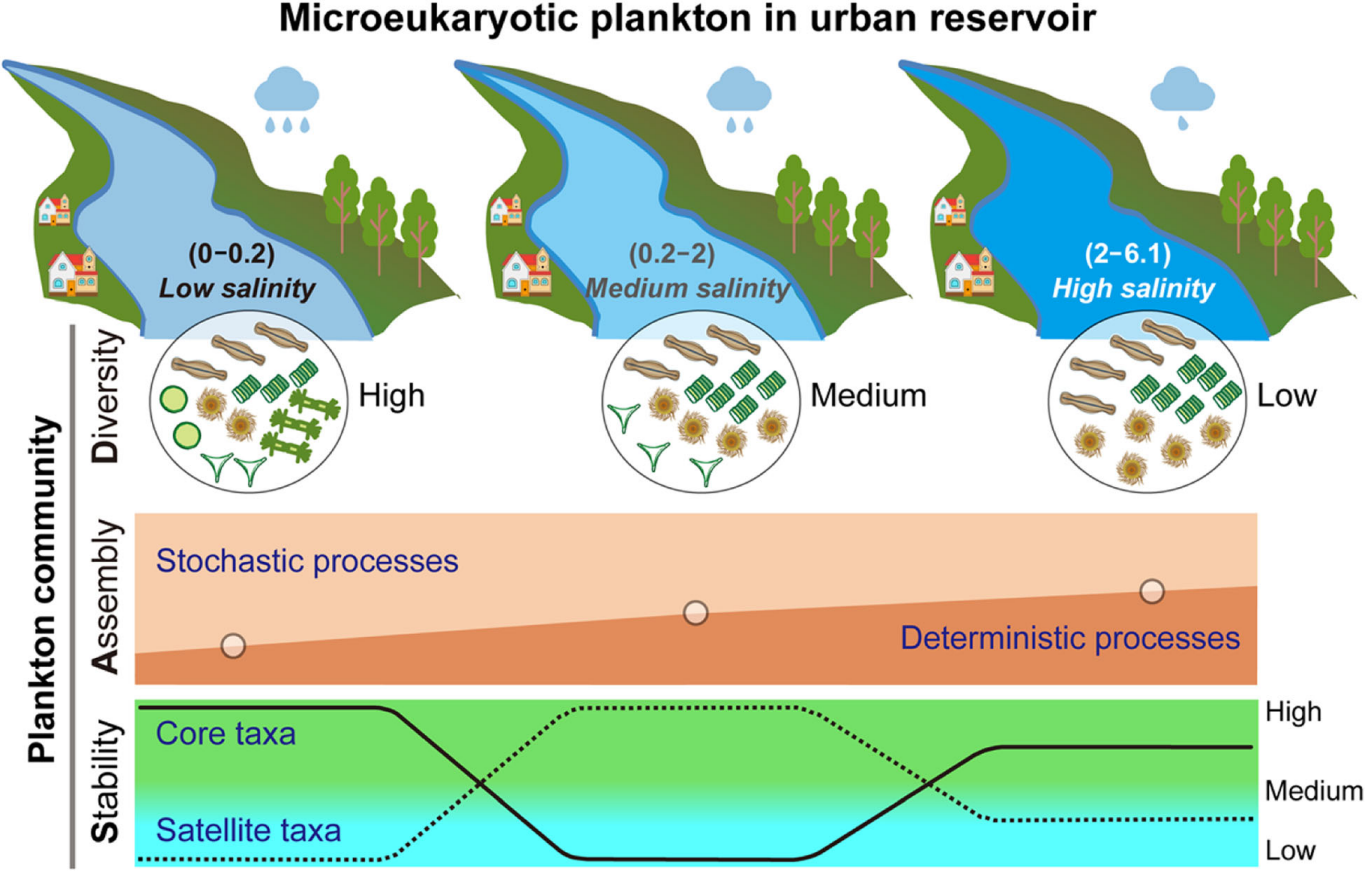
Conceptual models of microeukaryotic plankton diversity, community assembly processes, and network stability driven by low shifts in salinity

We propose a conceptual framework to describe the microeukaryotic plankton community responses to low shifts in salinity in inland freshwaters (Fig. 6). Low increases in salinity decrease the alpha-diversity of microeukaryotic plankton communities. All, core, and satellite plankton communities show the strongest relationship with salinity, followed by temperature and bacterial diversity or community due to microbial interactions or synchronous dynamics. Importantly, our results help unraveling the mechanisms affecting the balance between the deterministic and stochastic assembly of microeukaryotic plankton with changes in salinity. The potential keystone species in the all network belong to the core taxa, and the beta-diversity of satellite subcommunities significantly affects multi-nutrient cycling, implying that core and satellite OTUs play important but different roles in maintaining ecological function. In addition, core plankton networks are more stable in low-salinity environments, whereas satellite networks are more stable in the medium-/high-salinity environments. Given that a low increase in salinity in this freshwater reservoir significantly influenced the plankton community, management and protection require better knowledge of the response of the plankton community to salinity changes and their interactions with other human or natural disturbances [3, 4, 58], when evaluating, modeling, and predicting salinity effects on coastal urban freshwater ecosystems.

## Methods

### Study station, sampling, and environmental information

Surface water samples were collected in Xinglinwan Reservoir, Xiamen City, Fujian Province, Southeast China, at three stations (station C: 24° 36′ 53′′ N, 118° 03′ 11′′ E; station L: 24° 36′ 21′′ N, 118° 03′ 37′′ E; station G: 24° 36′ 09′′ N, 118° 03′ 59′′ E) (Fig. 1a). Specifically, samples were collected approximately daily from August 12, 2016 to August 30, 2016 at station C (12 samples), daily or twice a week from August 12 to September 20 in 2016 at station L (22 samples), and approximately daily from August 12 to September 20, 2016 and then twice a week from September 23, 2016 to August 18, 2017 at station G (116 samples). Each water sample was divided into two subsamples: one for microeukaryotic plankton analyses and the other for water chemistry analyses. About 500 mL of surface water (upper 50 cm) was filtered through a 200-μm mesh to remove larger particles and then filtered through 0.22-μm pore-size polycarbonate membrane filters (47-mm diameter, Millipore, Billerica, MA, USA) to collect the microeukaryotic cells within 60-min. The filters were then stored at – 80 °C until further analysis.

In addition, 18 environmental variables were measured or collected (Additional file 1: Figure S1 and S2). Water temperature, pH, dissolved oxygen, turbidity, electrical conductivity, salinity, and oxidation-reduction potential (ORP) were measured in situ with a Hydrolab DS5 multiparameter water quality analyzer (Hach Company, Loveland, CO, USA). Chl-*a* concentrations were quantified by PHYTO-PAM Phytoplankton Analyzer (Heinz Walz GmbH, Eichenring, Germany). Total carbon (TC), total organic carbon (TOC), total nitrogen (TN), ammonium nitrogen (NH_4_-N), nitrate nitrogen (NO_3_-N), nitrite nitrogen (NO_2_-N), total phosphorus (TP), and phosphate phosphorus (PO_4_-P) were measured according to the standard methods described in our previous study [59]. Precipitation and daily average wind speed data were downloaded from the Xiamen Meteorological Bureau. The precipitation data consisted of a 7-day accumulation before the sampling day. To study the relationship between salinity and precipitation, data on daily precipitation were collected and 3-year salinity data were measured from 2016 to 2018 (Additional file 1: Figure S3).

### DNA extraction, PCR, and Illumina sequencing

The total DNA was extracted from the filters using the FastDNA SPIN Kit and the FastPrep Instrument (MP Biomedicals, Santa Ana, CA, USA) following the manufacturer’s instructions. For the microeukaryotic plankton community, the V9 region of eukaryotic 18S rRNA gene was amplified using the primer pair 1380F and 1510R [60]. The 30-μL PCR reaction included 15-μL of Phusion High-Fidelity PCR Master Mix (New England Biolabs, Beverly, MA, USA), 0.2 μM of each primer, and 10 ng of community DNA. The PCR reactions included initial denaturation at 98 °C for 1 min, followed by 30 cycles of 10 s at 98 °C, 50 °C for 60 s, and 72 °C for 30 s. At the end of the amplification, the amplicons were subjected to a final 10 min extension at 72 °C. The PCR products from triplicate reactions per sample were pooled and gel-purified. All libraries were sequenced on the Illumina HiSeq platform (Illumina Inc., San Diego, CA, USA) using a paired-end (2 × 150 bp) approach.

To explore whether bacterial communities had an impact on the microeukaryotic plankton community, the V3-V4 hypervariable regions of the 16S rRNA gene were amplified, purified, and quantified following our previous procedure [61]. The triplicate PCR products were pooled together, and sequencing was performed on the Illumina MiSeq platform (Illumina, Inc., San Diego, CA, USA) using 2 × 250 bp paired-end sequencing approach.

### Bioinformatics

Pairs of reads from both the 18S rRNA and 16S rRNA gene were processed using VSEARCH v.2.14.1 [62]. Quality check and sequence merge were conducted using MOTHUR v.1.39.5 [63], and the filtered reads (“sequences”) were then processed as unique sequences using “minuniquesize 8” parameter in VSEARCH. The unoise3 algorithm was used to discard chimeras and assign operational taxonomic units (OTUs) at a 97% sequence similarity threshold in USEARCH v11 [64]. Subsequently, for microeukaryotic plankton, representative sequences from each OTU were taxonomically classified using an 80% confidence threshold against the Protist Ribosomal Reference (PR2) reference sequence database [65]. To make the taxonomic classification more user friendly and portable, the taxonomic assignments were adjusted to be in accordance with the taxonomic reference of eukaryotes [66, 67]. To minimize inclusion of sequencing errors, OTUs present in < 5 samples with < 10 sequences were excluded from the downstream analyses. After the OTU table was generated, we randomly rarefied a subset of 146,973 sequences from each of the 150 samples to standardize the sequencing effort using MOTHUR v.1.39.5 [63]. The 146,973 sequences were selected because they represented the sample with the lowest sequence number from all the samples. Finally, the total dataset retained 19,952 OTUs and 22,045,950 sequences at 97% similarity threshold. OTU numbers of unclassified Eukaryota accounted for 19.2% of the whole OTUs, and sequences of unclassified Eukaryota accounted for 3.3% of the whole sequences. For bacterial communities, OTU sequences were taxonomically classified by running USEARCH v11 against the Greengenes database [68]. All archaea, chloroplasts, eukaryota, mitochondria, and unknown sequences were discarded. Bacterial OTUs present in < 5 samples with < 10 sequences were removed. Finally, the total dataset was randomly normalized to 52,248 sequences for each of 116 samples from station G, and these sequences were clustered into 16,153 OTUs at a 97% similarity threshold.

Considering that the microeukaryotic OTUs identified at the 97% similarity level with the unoise3 algorithm is not a specific and accurate estimation of the species or strain level diversity, we further defined ASVs (amplicon sequence variants) using the unoise3 algorithm, as described previously [69]. Reads were quality-filtered to a maximum expected error threshold of 1.0, and then unoise3 was performed to identify ASVs using default settings dataset. In this study, ASVs were included only for beta-diversity analysis of microeukaryotic plankton to assess simply whether our results were biased by the OTUs definition approach.

### Real-time quantitative PCR

Real-time quantitative PCR (qPCR) was used to quantify the number of microeukaryotic plankton 18S rRNA gene copies using a LightCycler 480 instrument (Roche, Basel, Switzerland). The 20-μL reaction mixture consisted of 10 μL 2 × LightCycler 480 SYBR Green I Master Mix (Roche, Basel, Switzerland), 2 μL DNA template, 0.8 μM of each primer, and 6.4 μL RNase-free water. The PCR runs included tested samples and a negative control in triplicate. The following thermal cycling conditions were used: 30 s at 94 °C, followed by 40 cycles of 5 s at 94 °C, 15 s at 50 °C, and 10 s at 72 °C. Gene fragments were diluted (10^8^−10^2^ gene copies/μL) to generate the standard curve using a plasmid containing the 18S rRNA gene. The amplification efficiency (E) of qPCR was calculated using the equation E = [10^(−1/slope)^ − 1]. The qPCR efficiency of 18S rRNA ranged from 85 to 108% in this study. In addition, qPCR amplification of bacterial 16S rRNA gene was also performed using a Lightcycler 480 instrument (Roche, Basel, Switzerland) according to our previous method [70]. The qPCR efficiency of 16S rRNA gene was between 95 and 105%.

### Definition of core and satellite taxa

Partitioning microbial communities into core and satellite taxa according to their abundance and occurrence frequency has contributed to our understanding of community assembly and functioning in many spatiotemporal datasets [32, 71]. We arbitrarily defined “core” and “satellite” taxa based on previous study [33]; thus, core taxa were defined as the OTUs with an occurrence frequency ≥ 75% in all samples and satellite taxa as the OTUs with an occurrence frequency < 50% in all samples. Detailed descriptions of core and satellite datasets are presented in Additional file 1: Figure S4 and Additional file 1: Table S2.

### Statistical analyses

Alpha-diversity index (i.e., Shannon-Wiener index) and Tukey’s honestly significant difference (Tukey HSD) post hoc test were conducted using R software (version 3.6.1) [72]. The alpha-diversity index was calculated for each sample using the diversity function in the “vegan” package [72]. Temporal and salinity effects on alpha-diversity were evaluated using two-way analysis of variance (two-way ANOVA).

Microeukaryotic plankton community composition was visualized using non-metric multidimensional scaling (NMDS) based on Bray-Curtis dissimilarities. Analysis of similarity (ANOSIM) was used to evaluate differences in microeukaryotic plankton communities between groups. To reveal the temporal dynamics of microeukaryotic plankton communities, a time-lag regression analysis was applied to analyze the Bray-Curtis dissimilarity between each pair of samples, and the time difference (time-lag) was then plotted against the community dissimilarity. The effects of time and salinity on the Bray-Curtis dissimilarity were evaluated using Spearman’s rank correlation. In this study, we used two types of time: the absolute time span and the annual cycle time span. For example, the time lag between January and December should be (i.e., from January to December) 1 rather than 12 in the second time type.

In order to study the influence of abiotic (environmental factors) and biotic (bacterial community) variables on microeukaryotic plankton community composition, we computed the pairwise Bray-Curtis distances between samples on the basis of the relative abundance of microeukaryotic plankton (the compositional data). We also computed the pairwise Euclidean distance between the samples on the basis of environmental data and bacterial alpha- and beta-diversity. Then, partial Mantel test [73] was performed to assess the correlation between microeukaryotic plankton community composition and environmental variables or bacterial data, respectively.

Further, to determine the relative contribution of time (in this study: month) and salinity to the assembly of microeukaryotic plankton communities, Mantel and partial Mantel tests were applied [73]. The similarity matrices of the microeukaryotic plankton community were generated based on the Bray-Curtis index. The time and salinity matrices were obtained using the Euclidean distance. Mantel test assessed the correlation between microeukaryotic plankton community (Bray-Curtis dissimilarity) and salinity (Euclidean distance) or time (Euclidean distance), respectively. The partial Mantel test was performed to estimate the relative contribution of salinity or time variables to the changes in the microeukaryotic plankton community.

In addition, to further study the roles of core and satellite taxa on ecosystem functions, we calculated the multi-nutrient cycling index that can track the cycling of multiple nutrients in aquatic ecosystem [74] (see Additional file 1 for a detailed description). Afterwards, random forest (RF) machine learning [75] was used to assess the effects of alpha- and beta-diversity of core and satellite subcommunities on the multi-nutrient cycling index (see Additional file 1 for detailed description).

### Neutral community model

To estimate the effects of stochastic processes on the microeukaryotic plankton community assembly, a neutral community model was used [76], applying non-linear least-squares to generate the best fit between the frequency of OTUs occurrence and their relative abundance [77]. *R*^2^ value indicates the goodness of fit to the model, which was calculated following the “Östman’s method” [78]. When *R*^2^ is close to 1, the community assembly is fully consistent with stochastic processes. When it does not describe the community composition, *R*^2^ can be ≤ 0. Model computations were performed with R version 3.6.1 [72].

### Habitat niche breadth

To explore the relative effects of stochastic and deterministic processes on microeukaryotic plankton communities, we calculated Levins’ niche breadth (B) index for the microeukaryotic plankton using the formula:

$$ {B}_j=\frac{1}{\sum_{i=1}^N{P_{ij}}^2} $$

where *B*_*j*_ indicates the habitat niche breadth of OTU _*j*_ in a metacommunity; *N* represents the total number of communities in each metacommunity; *P*_*ij*_ is the proportion of OTU *j* in community *i* [23, 47]. A given OTU with high B value represents a wide habitat niche breadth. The community level B value (*Bcom*) was calculated as the average of B values from all taxa occurring in one given community [23, 49]. A microeukaryotic plankton community with a wide niche breadth is expected to be metabolically more flexible at the community level than one with a narrow niche breadth [23, 47, 49]. The analysis was performed using the “niche.width” function within R package “spaa” [79].

### Null model

We tested clustering or overdispersion of microeukaryotic plankton communities by examining the deviation of each observed metric from the average of the null model (checkerboard score (C-score)) [80]. The values obtained were standardized to allow comparisons among assemblages using the standardized effect size (SES). Specifically, the sequence table was transformed into a binary matrix of presence (1) and absence (0), and then SES was calculated under the null model [81]. The SES for C-score was estimated as the difference between the observed index and the mean of the stimulated index over the standard deviation of the stimulated index [82]. The higher or lower SES value than the expected null value is interpreted as overdispersion or underdispersion, respectively, and the magnitude of SES is interpreted as the strength of the effect of deterministic processes on the assemblage [83]. C-score was evaluated based on 30,000 simulations and using the sequential swap randomization algorithm with the package “EcoSimR” in R version 3.6.1 [72].

### Network construction

We constructed species co-occurrence networks based on samples from the whole study period (August 2016–August 2017, 116 samples) for station G in the Xinglinwan Reservoir. To reduce the complexity of the datasets, we removed OTUs present in less than 20 samples with less than 200 sequences for the construction of networks. We also constructed community subnetworks for the all, core, and satellite microeukaryotic plankton based on samples at the three salinity levels, respectively.

SparCC was used to calculate pairwise correlations between plankton OTUs [84]. Only robust (|r| > 0.6) and statistically significant (*P* < 0.01) correlations were incorporated into the network analyses. Network visualization was generated with Cytoscape version 3.6.1 and Gephi version 0.9.1. Each node indicates a given OTU, and each edge represents a significant correlation between two OTUs. Degree represents the number of edges connecting each node to the rest nodes of the network. Normally, the high topological characteristic values (such as node, edge, and degree) suggest a more complex network. In general, there are two common network distributions. One is the random network. A random network follows a Poisson distribution of edges per node, meaning that there are no highly associated nodes and that most nodes exhibit a similar number of edges [85]. The other is non-random network. That is, scale-free or small-world network that has a power-law distribution, implying that some nodes are highly associated and maintaining the network together [86, 87].

Based on metabolic network approaches [88], the network hubs (Zi-score > 2.5; Pi-score > 0.62), module hubs (Zi-score > 2.5; Pi-score < 0.62), connectors (Zi-score < 2.5; Pi-score > 0.62), and peripherals (Zi-score < 2.5; Pi-score < 0.62) were identified [87]. All hubs and connector nodes could be defined as potential keystone species in co-occurrence networks [89]. Furthermore, the 1000 Erdös-Réyni random networks, which exhibit the same number of nodes and edges as the real networks, were calculated in the “igraph” R package, with each edge having the same probability of being assigned to a node [85]. To further describe the topological parameters of the networks, a set of metrics of both real and random networks were calculated and compared: clustering coefficient, average path length, and modularity.

The network dissimilarity between different salinity levels was identified using the widely applied equation, which consists of a re-expression of classical measures of dissimilarity following a partition of shared and total items [90, 91]:

$$ {\beta}_w=\frac{a+b+c}{\left(2a+b+c\right)/2}-1 $$

where *βw* is dissimilarity between networks B and C, *a* represents number of shared edges between networks B and C, *b* represents number of edges unique to network B, and *c* represents number of edges unique to network C.

Finally, network stability was evaluated by removing nodes in the static network to estimate how quickly robustness degraded, and network robustness was assessed by natural connectivity of the nodes. The node removing was a random repetitive process [92].

## Supplementary Information

**Additional file 1: Figure S1.** Temporal dynamics of 18 major environmental factors in stations C, L, and G from Xinglinwan Reservoir from August 12, 2016 to September 20, 2016. **Figure S2.** Temporal dynamics of 18 major environmental factors in station G from Xinglinwan Reservoir from August 2016 to August 2017. **Figure S3.** Changes of precipitation and salinity over three years. **Figure S4.** Definition of core, intermediate, and satellite plankton in the microeukaryotic metacommunity in Xinglinwan Reservoir. **Figure S5.** Temporal dynamic of microeukaryotic plankton communities. **Figure S6.** The absolute abundance of microeukaryotic plankton and bacterioplankton from stations C, L, and G in Xinglinwan Reservoir. **Figure S7.** Variability of microeukaryotic plankton communities across three salinity levels from the three stations of Xinglinwan Reservoir. **Figure S8.** Community structuring of microeukaryotic plankton across salinity gradient and time series at station G. **Figure S9.** Comparison of community composition of all, core and satellite microeukaryotic plankton from station G among three salinity levels at phylum level. **Figure S10.** Opposite changes in stochasticity and salinity during the community succession of microeukaryotic plankton. **Figure S11.** Comparison of mean niche breadth between core and satellite subcommunities at station G (*n* = 116). **Figure S12.** The potential importance of core and satellite microeukaryotic plankton in community network and nutrient cycle, respectively. **Table S1.** Previous literatures analyzing the influence of salinity changes on plankton or microorganism. **Table S2.** Description of microeukaryotic plankton OTUs datasets at 97% sequence similarity level. **Table S3.** Mantel and partial Mantel tests showing the relationship between microeukaryotic plankton community similarity and salinity and time (month) using Pearson’s coefficient. **Table S4.** Topological properties of the empirical species co-occurrence networks of microeukaryotic plankton communities and their associated random network. **Table S5.** Number of degrees of different microeukaryotic plankton groups in six different modules in integrated networks of station G. **Table S6.** Numbers of shared OTUs (or nodes) and unique edges (correlations) and their dissimilarity between different microeukaryotic plankton sub-networks based on the three different salinity levels.

**Additional file 2.**

## Data Availability

All raw sequences from this study have been stored in the public NCBI Sequence Read Archive (SRA) database under the BioProject number PRJNA510458 and the accession number SRP173869 for 18S rRNA gene, and under BioProject number PRJNA510463 and accession number SRP173857 for 16S rRNA gene.
