# Supplementary material for: Low shifts in salinity determined assembly processes and network stability of microeukaryotic plankton communities in a subtropical urban reservoir
Source: Microbiome. 2021 Jun 3;9:128. doi: 10.1186/s40168-021-01079-w (PMC8176698; doi:10.1186/s40168-021-01079-w)
Supplement: Supplementary file 2 — Additional file 1: Figure S1. Temporal dynamics of 18 major environmental factors in stations C, L, and G from Xinglinwan Reservoir from August 12, 2016 to September 20, 2016. Figure S2. Temporal dynamics of 18 major environmental factors in station G from Xinglinwan Reservoir from August 2016 to August 2017. Figure S3. Changes of precipitation and salinity over three years. Figure S4. Definition of core, intermediate, and satellite plankton in the microeukaryotic metacommunity in Xinglinwan Reservoir. Figure S5. Temporal dynamic of microeukaryotic plankton communities. Figure S6. The absolute abundance of microeukaryotic plankton and bacterioplankton from stations C, L, and G in Xinglinwan Reservoir. Figure S7. Variability of microeukaryotic plankton communities across three salinity levels from the three stations of Xinglinwan Reservoir. Figure S8. Community structuring of microeukaryotic plankton across salinity gradient and time series at station G. Figure S9. Comparison of community composition of all, core and satellite microeukaryotic plankton from station G among three salinity levels at phylum level. Figure S10. Opposite changes in stochasticity and salinity during the community succession of microeukaryotic plankton. Figure S11. Comparison of mean niche breadth between core and satellite subcommunities at station G (n = 116). Figure S12. The potential importance of core and satellite microeukaryotic plankton in community network and nutrient cycle, respectively. Table S1. Previous literatures analyzing the influence of salinity changes on plankton or microorganism. Table S2. Description of microeukaryotic plankton OTUs datasets at 97% sequence similarity level. Table S3. Mantel and partial Mantel tests showing the relationship between microeukaryotic plankton community similarity and salinity and time (month) using Pearson’s coefficient. Table S4. Topological properties of the empirical species co-occurrence networks of microeukaryotic plankton communities [file 40168_2021_1079_MOESM2_ESM.pdf]

Journal: Microbiome

Additional file 1 for:

Low shifts in salinity determined assembly processes and network stability of microeukaryotic plankton communities in a subtropical urban reservoir

Yuanyuan Mo[1,2], Feng Peng[1], Xiaofei Gao[1,2], Peng Xiao[1], Ramiro Logares[3], Erik Jeppesen[4,5,6,7], Kexin Ren[1], Yuanyuan Xue[1], Jun Yang[1,*]

[1] *Aquatic Ecohealth Group, Fujian Key Laboratory of Watershed Ecology, Key Laboratory of Urban Environment and Health, Institute of Urban Environment, Chinese Academy of Sciences, Xiamen 361021, China*

[2] *University of Chinese Academy of Sciences, Beijing 100049, China*

[3] *Institute of Marine Sciences, CSIC, Passeig Marítim de la Barceloneta 37-49, Barcelona, ES08003, Spain*

[4] *Department of Bioscience, Aarhus University, Silkeborg 8600, Denmark*

[5] *Sino-Danish Centre for Education and Research, Beijing 100049, China*

[6] *Limnology Laboratory, Department of Biological Sciences and Centre for Ecosystem Research and Implementation, Middle East Technical University, Ankara 06800, Turkey*

[7] *Institute of Marine Sciences, Middle East Technical University, Erdemli-Mersin 33731, Turkey*

***Correspondence author:**

Jun Yang, E-mail address: jyang@iue.ac.cn

This supplementary information contains:

- 23 Pages
- 12 Figures
- 6 Tables
- 19 References

This file includes:

Figure S1. Temporal dynamics of 18 major environmental factors in stations C, L, and G from Xinglinwan Reservoir from August 12, 2016 to September 20, 2016.

Figure S2. Temporal dynamics of 18 major environmental factors in station G from Xinglinwan Reservoir from August 2016 to August 2017.

Figure S3. Changes of precipitation and salinity over three years.

Figure S4. Definition of core, intermediate, and satellite plankton in the microeukaryotic metacommunity in Xinglinwan Reservoir.

Figure S5. Temporal dynamic of microeukaryotic plankton communities.

Figure S6. The absolute abundance of microeukaryotic plankton and bacterioplankton from stations C, L, and G in Xinglinwan Reservoir.

Figure S7. Variability of microeukaryotic plankton communities across three salinity levels from the three stations of Xinglinwan Reservoir.

Figure S8. Community structuring of microeukaryotic plankton across salinity gradient and time series at station G.

Figure S9. Comparison of community composition of all, core and satellite microeukaryotic plankton from station G among three salinity levels at phylum level.

Figure S10. Opposite changes in stochasticity and salinity during the community succession of microeukaryotic plankton.

Figure S11. Comparison of mean niche breadth between core and satellite subcommunities at station G (n = 116).

Figure S12. The potential importance of core and satellite microeukaryotic plankton in community network and nutrient cycle, respectively.

Table S1. Previous literatures analyzing the influence of salinity changes on plankton or microorganism.

Table S2. Description of microeukaryotic plankton OTUs datasets at 97% sequence similarity level.

Table S3. Mantel and partial Mantel tests showing the relationship between microeukaryotic plankton community similarity and salinity and time (month) using Pearson's coefficient.

Table S4. Topological properties of the empirical species co-occurrence networks of microeukaryotic plankton communities and their associated random network.

Table S5. Number of degrees of different microeukaryotic plankton groups in six different modules in integrated networks of station G.

Table S6. Numbers of shared OTUs (or nodes) and unique edges (correlations) and their dissimilarity between different microeukaryotic plankton sub-networks based on the three different salinity levels.

Method

Multi-nutrient cycling index

The multi-nutrient cycling index was analyzed to track the cycling of multiple nutrients in aquatic ecosystems (Gao et al., 2021). It is similar to the multi-functionality index for nutrient cycling in terrestrial ecosystems (Maestre et al., 2012; Delgado-Baquerizo et al., 2016; Jiao et al., 2019). We calculated the multi-nutrient cycling index using eight measured nutrient properties: namely total carbon (TC), total organic carbon (TOC), total nitrogen (TN), ammonium nitrogen (NH_4-N), nitrate nitrogen (NO_3-N), nitrite nitrogen (NO_2-N), total phosphorus (TP) and phosphate phosphorus (PO_4-P). To obtain the multi-nutrient cycling index for each sample, we first normalized (log (x+1) transformed) and standardized each of the eight nutrient parameters by Z-score transformation. The standardized functions were then averaged to get the multi-nutrient cycling index.

Random forest machine learning

Random forest (RF) is a machine learning algorithm used for regression and classification (Breiman et al., 2001). In this study, it was used to assess the effect of alpha- and beta-diversity of core and satellite plankton subcommunities on multi-nutrient cycling index. On the one hand, RF analysis can effectively handle data with some missing data. On the other hand, the analysis is unlike traditional regression tree analysis, because it can assess the fit of each tree with the prediction error. The variables included in the model leading to the smallest prediction error were selected as important predictors (Genuer et al., 2010). Thus, RF exhibits high accuracy and is a very efficient algorithm, based on model aggregation ideas. The model significance was estimated using 5000 permutations of the response variable with the “A3” (Fortmann-Roe, 2015) R package. The significance of each predictor on the response variable (multi-nutrient cycling index) was evaluated with the “rfPermute” (http://cran. rproject.org/web/packages/rfPermute/rfPermute.pdf) R package.

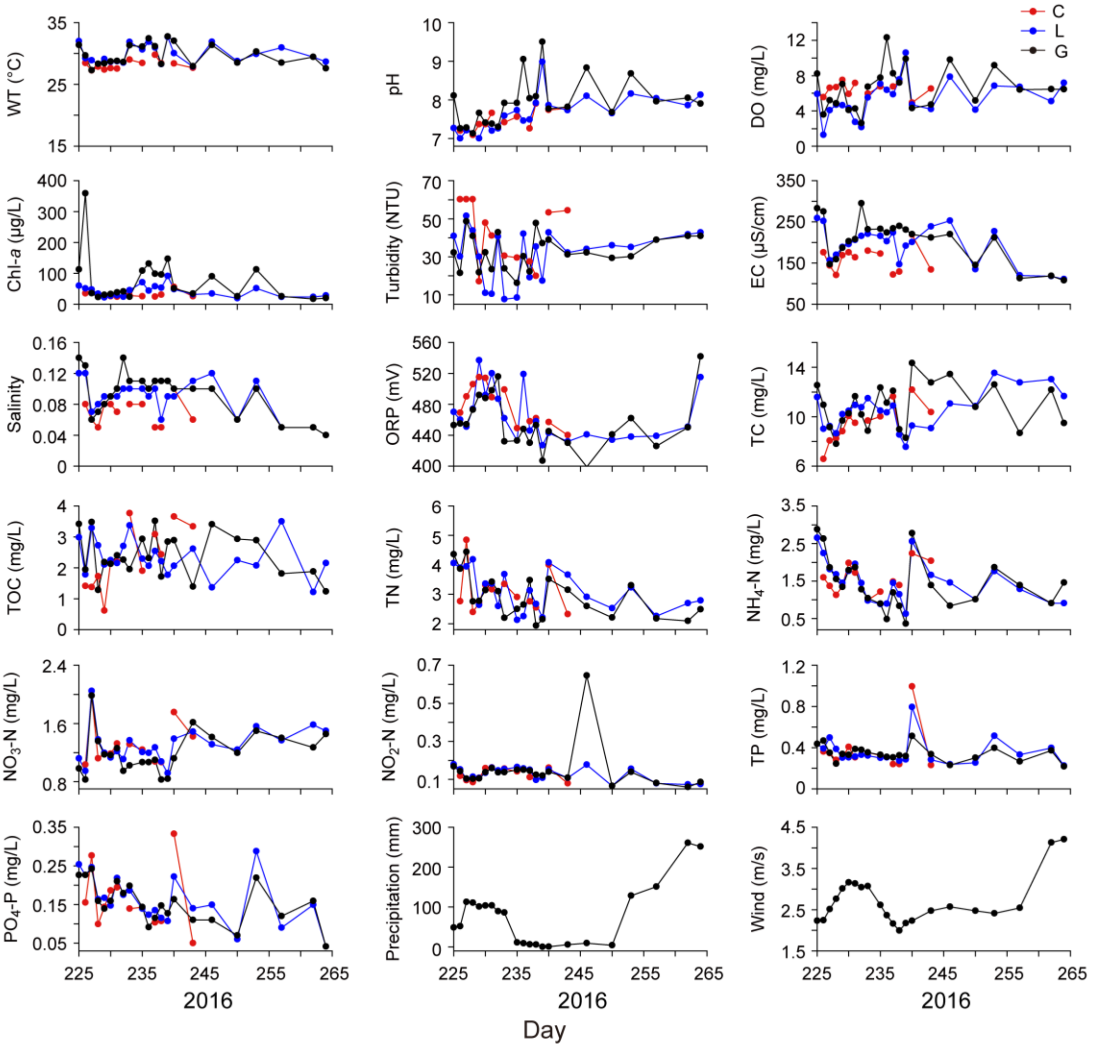

Figure S1. Temporal dynamics of 18 major environmental factors in stations C, L, and G from Xinglinwan Reservoir from August 12, 2016 to September 20, 2016. WT, water temperature; DO, dissolved oxygen; Chl-*a*, chlorophyll-*a*; EC, electrical conductivity; ORP, oxidation-reduction potential; TC, total carbon; TOC, total organic carbon; TN, total nitrogen; NH_4-N, ammonium nitrogen, NO_3-N, nitrate nitrogen; NO_2-N, nitrite nitrogen; TP, total phosphorus; PO_4-P, phosphate phosphorus. Note that the precipitation data are the 7-day accumulation before the sampling day, and the wind represents daily average wind speed.

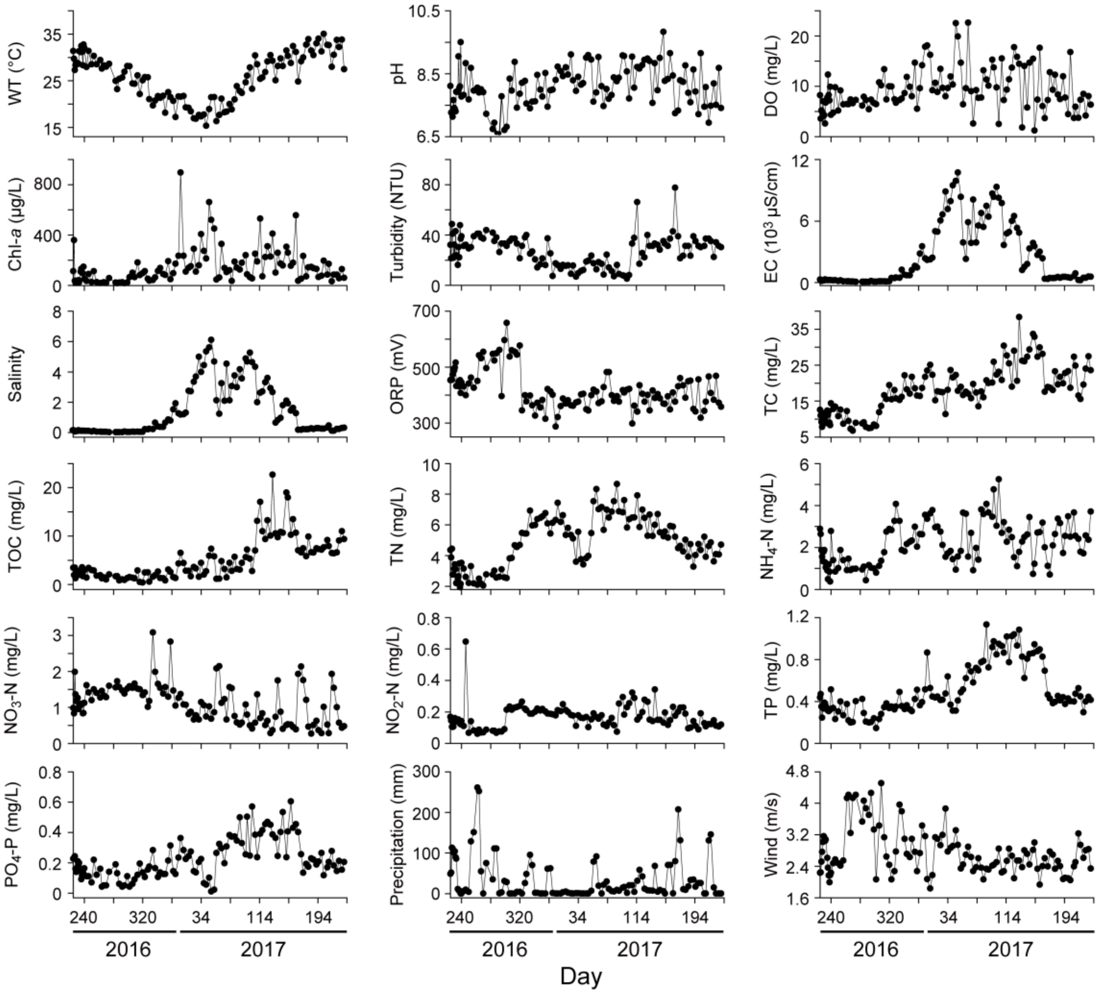

Figure S2. Temporal dynamics of 18 major environmental factors in station G from Xinglinwan Reservoir from August 2016 to August 2017. WT, water temperature; DO, dissolved oxygen; Chl-*a*, chlorophyll-*a*; EC, electrical conductivity; ORP, oxidation-reduction potential; TC, total carbon; TOC, total organic carbon; TN, total nitrogen; NH_4-N, ammonium nitrogen, NO_3-N, nitrate nitrogen; NO_2-N, nitrite nitrogen; TP, total phosphorus; PO_4-P, phosphate phosphorus. Note that the precipitation data are the 7-day accumulation before the sampling day, and the wind represents daily average wind speed.

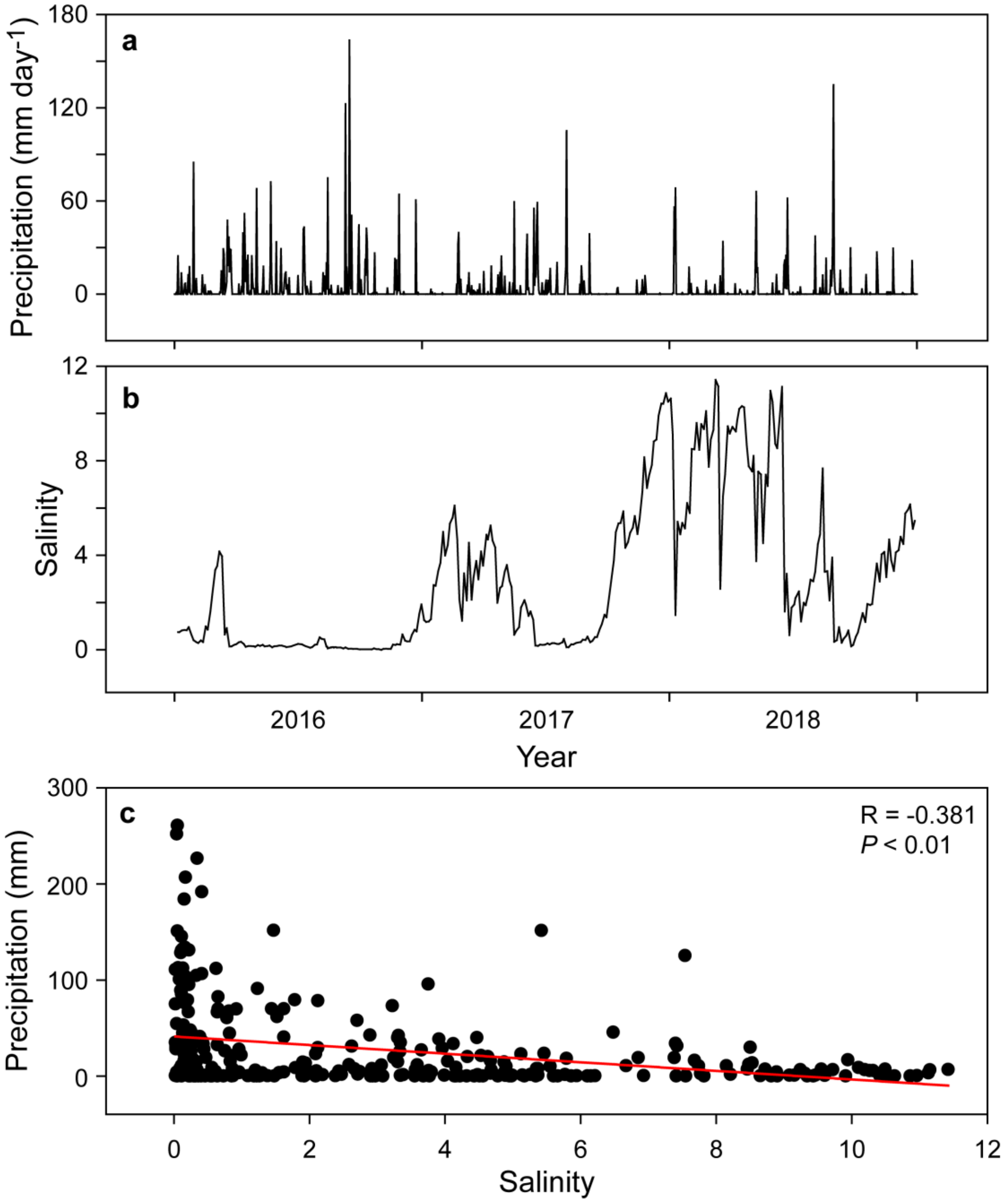

Figure S3. Changes of precipitation and salinity over three years. a, Daily precipitation from 2016 to 2018; **b**, Temporal dynamics of salinity in station G of Xinglinwan Reservoir from 2016 to 2018; **c**, Significant relationship between precipitation and salinity. The precipitation data are the 7-day accumulation before the sampling day. The coefficient R value and significant *P* value are derived from Spearman's correlation.

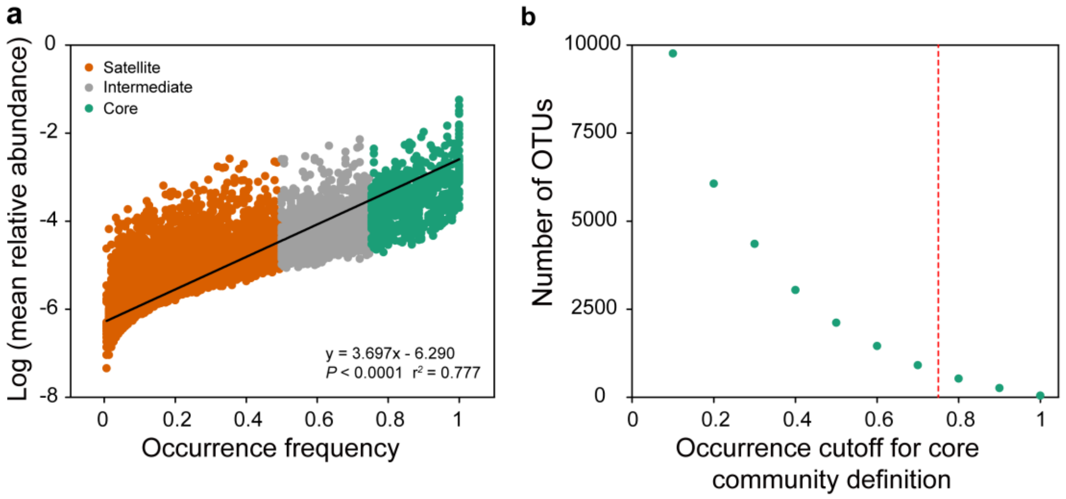

Figure S4. Definition of core, intermediate, and satellite plankton in the microeukaryotic metacommunity in Xinglinwan Reservoir. The mean relative abundance of OTUs (**a**) and the number of OTUs (**b**) are shown relative to the OTUs percentage occurrence. Core taxa were defined as the OTUs with an occurrence frequency ≥ 75% in all samples. Satellite taxa were defined as the OTUs with an occurrence frequency < 50% in all samples. The red dash line in figure **b** indicates the threshold of core OTUs (75%).

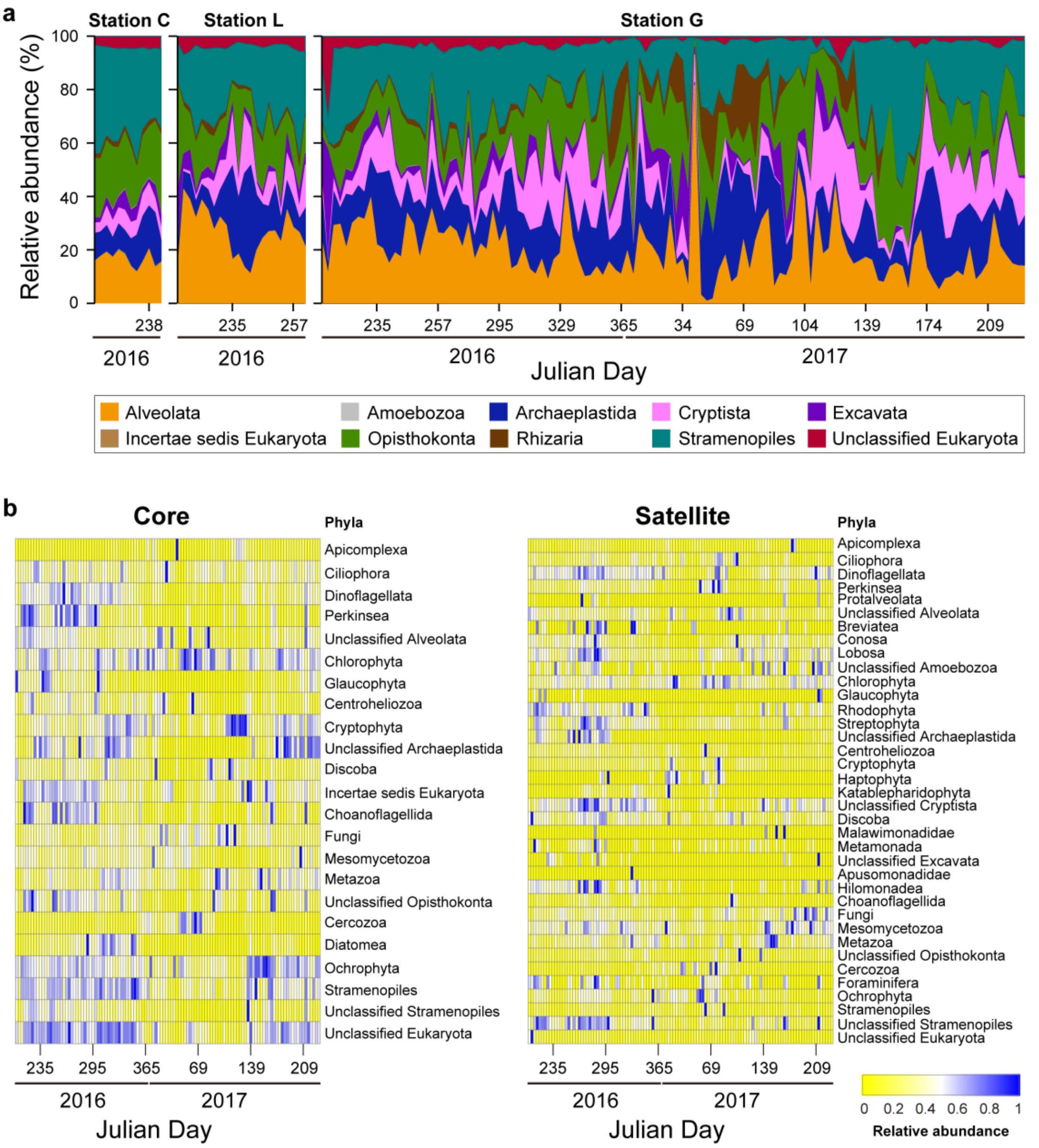

Figure S5. Temporal dynamic of microeukaryotic plankton communities. a, Change of microeukaryotic communities based on relative abundance datasets at the super-group level from stations C, L, and G in Xinglinwan Reservoir. **b**, A heat map revealing the distribution of the core and satellite taxa based on relative abundance across time at the phylum level from station G.

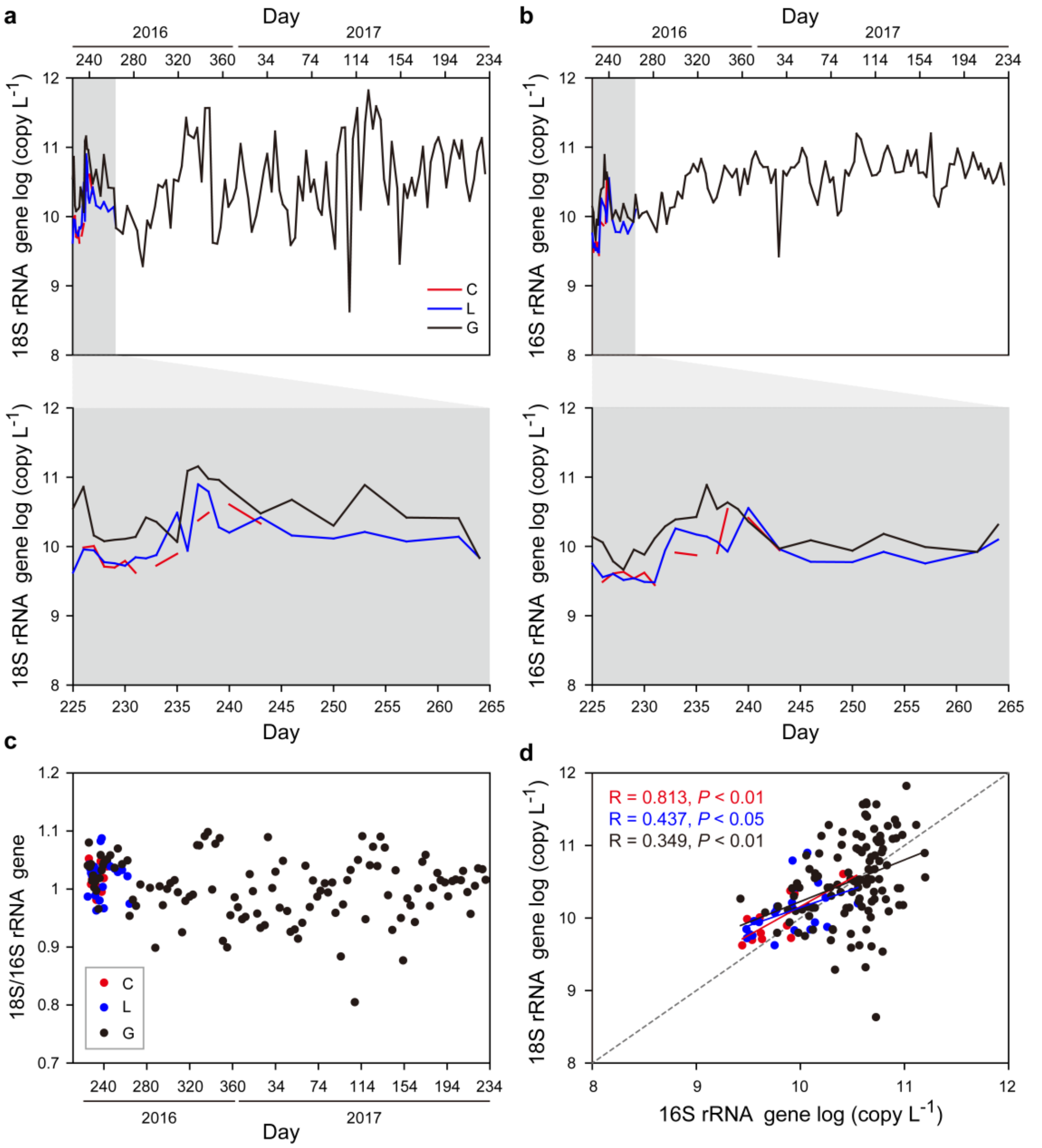

Figure S6. The absolute abundance of microeukaryotic plankton and bacterioplankton from stations C, L, and G in Xinglinwan Reservoir. The rRNA gene copy numbers are based on qPCR data. The figure shows changes in absolute abundance of microeukaryotic plankton (**a**) and bacterioplankton (**b**), and the consequent changes in ratio of microeukaryotic 18S rRNA gene to bacterial 16S rRNA gene across the time series (**c**). Also shown is the relationship between microeukaryotic plankton and bacterioplankton (**d**). The dash line in the **d** is the y = x line. Red, station C; blue, station L; black, station G.

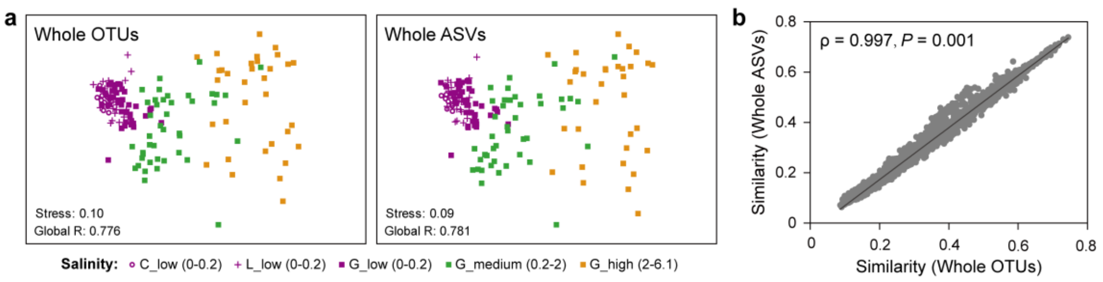

Figure S7. Variability of microeukaryotic plankton communities across three salinity levels from the three stations of Xinglinwan Reservoir. a, Non-metric multidimensional scaling (NMDS) ordination based on Bray-Curtis similarity showing the variation of microeukaryotic plankton communities across three salinity levels. Whole OTUs, whole microeukaryotic plankton OTUs datasets at 97% sequence similarity level including stations C, L, and G; Whole ASVs, whole microeukaryotic plankton ASVs datasets at 100% sequence similarity level including stations C, L, and G. Note that the significant level of whole OTUs and ASVs is $P = 0.001$ among three salinity levels. **b,** Spearman correlation of Bray-Curtis similarity (RELATE analysis) between the Whole OTUs and Whole ASVs. Correlations were calculated as pairwise comparisons of similarity matrix data; the ρ value indicates the correlation coefficient, and the black line is $y = x$.

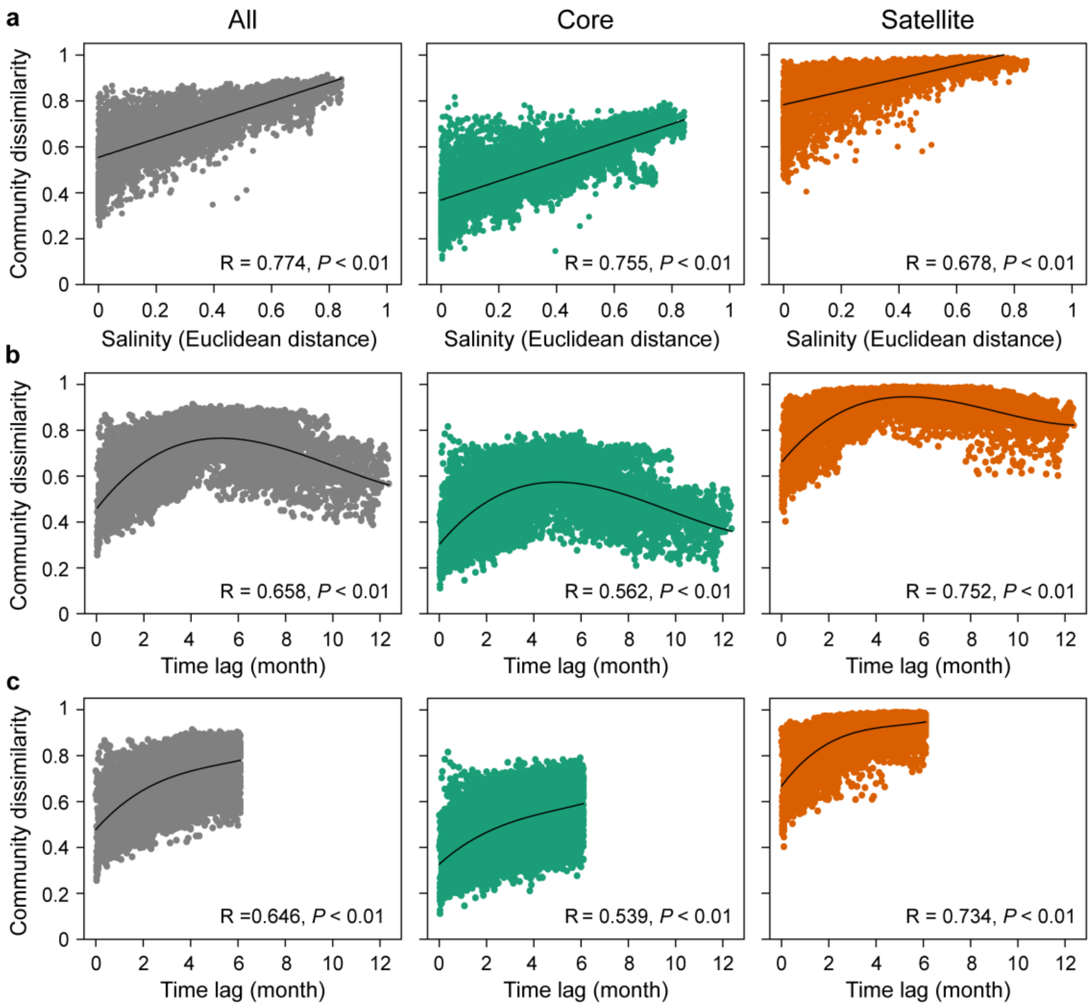

Figure S8. Community structuring of microeukaryotic plankton across salinity gradient and time series at station G. a, Correlation between salinity and Bray-Curtis dissimilarity of microeukaryotic communities. **b,** Time-lag regression analysis of Bray-Curtis dissimilarity for microeukaryotic communities based on absolute time span. **c,** Time-lag regression analysis of Bray-Curtis dissimilarity for microeukaryotic communities based on the annual cycle time span.

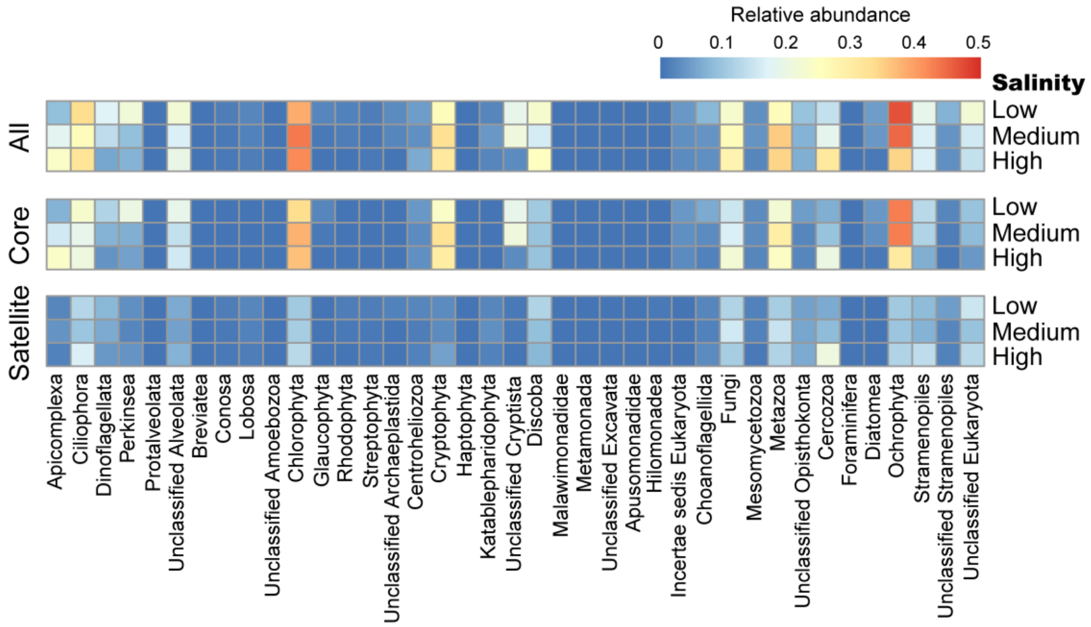

Figure S9. Comparison of community composition of all, core and satellite microeukaryotic plankton from station G among three salinity levels at phylum level. Each cell represents the square root transformed mean relative abundance of each taxonomic group at each salinity level.

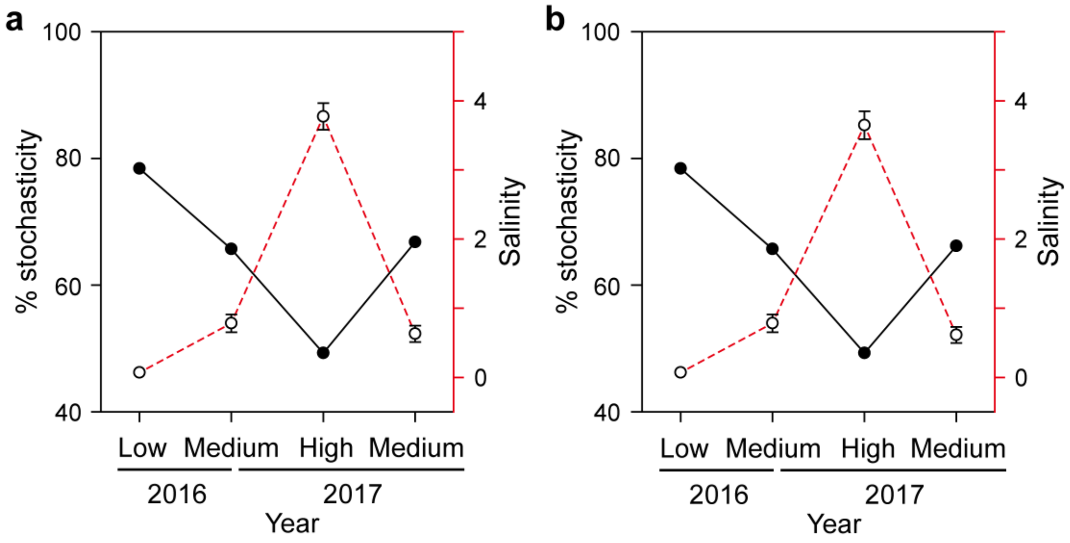

Figure S10. Opposite changes in stochasticity and salinity during the community succession of microeukaryotic plankton. Note that the same results were obtained from datasets where outliers (for three salinity levels) were (**a**) not removed and (**b**) removed. Outliers represent samples that do not belong to the corresponding salinity level during the successional period. Salinity data are expressed as means ± standard error (error bars).

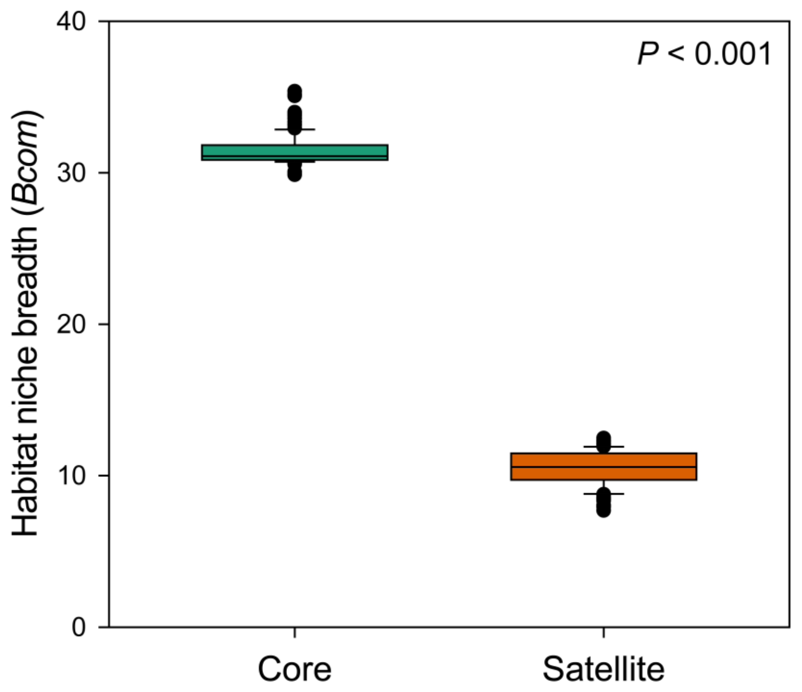

Figure S11. Comparison of mean niche breadth between core and satellite subcommunities at Station G (n = 116). The *Bcom* value was calculated using the mean niche breadth (*Bcom*) of all taxa in a sub-community. Statistical analysis is non-parametric Mann-Whitney *U* test. Core, core microeukaryotic plankton; Satellite, satellite microeukaryotic plankton.

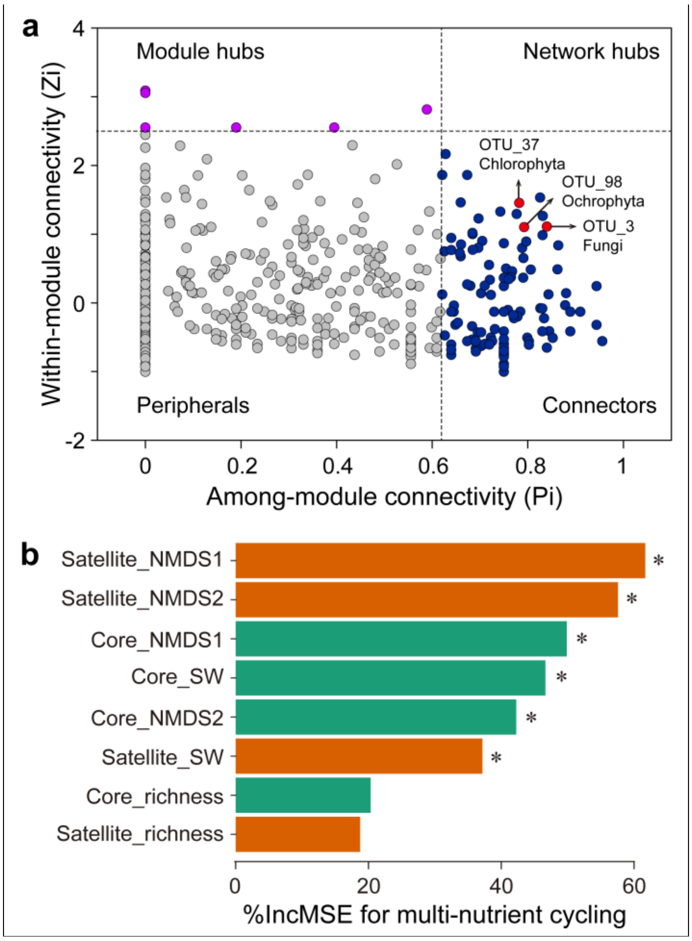

Figure S12. The potential importance of core and satellite microeukaryotic plankton in community network and nutrient cycle, respectively. a, Zi-Pi plot showing the distribution of all OTUs based on their topological properties. Each circle-dot symbol represents an OTU. The topological role of each OTU was determined according to the scatter plot of within-module connectivity (Zi) and among-module connectivity (Pi). The top three highest degrees of connectors are indicated by red circle-dots, indicating these three OTUs are very important in maintaining the network structure and stability. Both OTU_3 and OTU_37 belong to core microeukaryotic plankton, while OTU_98 belongs to intermediate microeukaryotic plankton. **b**, Random forest showing predictor importance of community diversity of core and satellite microeukaryotic plankton in the multi-nutrient cycling. NMDS1, NMDS ordination axis 1; NMDS2, NMDS ordination axis 2; richness, OTU number; SW, Shannon-Wiener index. The accuracy importance measure was computed for each tree and averaged over the forest (based on 5,000 trees). Significant level: * $P < 0.05$.

Table S1. Previous literatures analyzing the influence of salinity changes on plankton or microorganism

**Habitat**	**Taxa**	**Characteristic**	**Salinity range (‰)**	**Reference**
Water	Bacteria	Community composition, diversity, function, stability	From 3 to 12	Berga et al., 2017
Water	Bacteria	Community composition, diversity, growth rate	From 0.1 to 31	Campbell et al., 2013
Water	Bacteria (Cyanobacteria)	Community structure, composition	From 35 to 85	Green et al., 2008 (Add NaCl and KCl)
Water	Bacteria	Community composition, diversity	From 0 to 30.9	Herlemann et al., 2011
Water	Bacteria	Community diversity, network complexity	From <0.5 to >50	Ji et al., 2019
Water	Bacteria	Community composition	From 0 to 100	Logares et al., 2013
Water	Archaea and Bacteria	Community composition, diversity	From <1 to 344	Zhong et al., 2016
Water	Bacteria	Community composition, diversity, primary producer	From <1 to >29	Yue et al., 2019
Water	Bacteria (*Psychroflexus torquis*)	Population	From 17.5 to 70	Feng et al., 2013
Water	Eukaryotic plankton	Community composition, diversity	From 0 to >50	Wang et al., 2014
Sediments	Archaea (Bathyarchaeota)	Community structure, abundance, function	From 0.3 to 34.5	Zou et al., 2020
Sediments	Bacteria	Community composition, diversity	From 0.1 to 426.3	Yang et al., 2016

Note that salinity is a ratio and does not have physical units

Table S2. Description of microeukaryotic plankton OTUs datasets at 97% sequence similarity level

Category	OTU number (Percentage)	Sequence number (Percentage)
Whole OTUs	19,952	22,045,950
Core taxa	618 (3.1%)	14,423,537 (65.4%)
Satellite taxa	18,056 (90.5%)	3,211,348 (14.6%)
All OTUs-C	15,057 (75.5%)	1,763,676 (8.0%)
Core taxa-C	618 (3.1%)	1,079,890 (4.9%)
Satellite taxa-C	13,161 (66.0%)	355,206 (1.6%)
All OTUs-L	15,753 (79.0%)	3,233,406 (14.7%)
Core taxa-L	618 (3.1%)	2,057,240 (9.3%)
Satellite taxa-L	13,857 (69.5%)	492,414 (2.2%)
All OTUs-G	19,388 (97.2%)	17,048,868 (77.3%)
Core taxa-G	618 (3.1%)	11,286,407 (51.2%)
Satellite taxa-G	17,492 (87.7%)	2,363,728 (10.7%)

C, station C; L, station L; G, station G

Core taxa were defined as the OTUs with an occurrence frequency ≥ 75% in all samples

Satellite taxa were defined as the OTUs with an occurrence frequency < 50% in all samples

The values in parentheses indicate the percentage of OTU number or sequence of different categories (core and satellite) in whole community

Table S3. Mantel and partial Mantel tests showing the relationship between microeukaryotic plankton community dissimilarity and salinity and time (month) using Pearson's coefficient

Effects of	Controlling for	All taxa	Core taxa	Satellite taxa
Salinity		0.675**	0.649**	0.568**
Time		0.359**	0.327**	0.490**
Salinity	Time	0.648**	0.621**	0.530**
Time	Salinity	0.270**	0.228**	0.440**

Significances are tested based on 999 permutations

**$P < 0.01$

Table S4. Topological properties of the empirical species co-occurrence networks of microeukaryotic plankton communities and their associated random network

Network properties		All	All Low	All Medium	All High	Core Low	Core Medium	Core High	Satellite Low	Satellite Medium	Satellite High
Empirical network	Nodes	993	1757	1295	589	468	452	251	316	290	166
	Links	8387	29484	20045	5009	4509	4183	1604	755	1316	582
	Positive	7336	21784	14878	4350	3288	2690	1392	723	1283	517
		**(87.47%)**	**(73.88%)**	**(74.22%)**	**(86.84%)**	**(72.92%)**	**(64.31%)**	**(86.78%)**	**(95.76%)**	**(97.49%)**	**(88.83%)**
	Negative	1051	7700	5167	659	1221	1493	212	32	33	65
		(12.53%)	***(26.12%)***	***(25.78%)***	***(13.16%)***	***(27.08%)***	***(35.69%)***	***(13.22%)***	***(4.24%)***	***(2.51%)***	***(11.17%)***
	Diameter	12	10	11	11	15	10	10	9	9	12
	Density	0.015	0.019	0.024	0.029	0.041	0.041	0.051	0.015	0.031	0.042
	avgCC	0.538	0.496	0.484	0.498	0.496	0.454	0.486	0.389	0.456	0.514
	avgPL	3.759	3.492	3.480	3.634	3.867	3.406	3.549	4.031	3.522	4.095
	Modularity	0.527	0.424	0.373	0.529	0.444	0.390	0.450	0.735	0.517	0.627
	R^2 of power-law	0.972	0.965	0.989	0.957	0.939	0.951	0.907	0.964	0.974	0.891
Random network	$avgCC_r$	0.017	0.019	0.024	0.029	0.041	0.041	0.051	0.015	0.031	0.042
		(0.001)	(0.000)	(0.000)	(0.001)	(0.001)	(0.001)	(0.003)	(0.004)	(0.003)	(0.005)
	$avgPL_r$	2.738	2.497	2.442	2.567	2.392	2.408	2.447	3.832	2.802	2.825
		(0.001)	(0.000)	(0.000)	(0.001)	(0.001)	(0.001)	(0.003)	(0.023)	(0.005)	(0.011)
	Modularity	0.170	0.102	0.112	0.176	0.164	0.169	0.214	0.386	0.264	0.305
		(0.004)	(0.002)	(0.002)	(0.005)	(0.005)	(0.005)	(0.009)	(0.014)	(0.011)	(0.014)
	σ	23.025	18.564	14.212	12.161	7.443	7.831	6.571	26.087	11.667	8.550
		(0.796)	(0.234)	(0.197)	(0.407)	(0.212)	(0.241)	(0.339)	(7.170)	(1.035)	(1.096)

Low, low salinity; medium, medium salinity; high, high salinity

Number of OTUs with the correlation $|r| > 0.6$ and statistical significance ($P < 0.01$)

In the empirical network, the numbers in parentheses represent the proportion of positive correlations in the network, and the italic numbers represent the proportion of negative correlations

In random network, the numbers in parentheses represent the standard deviation

avgCC, average clustering coefficient; avgPL, average path length; r, random network; σ, small-world network characteristics

Table S5. Number of degrees of different microeukaryotic plankton groups in six different modules in integrated networks of station G

	M1	M2	M3	M4	M5	M6
Apicomplexa	110	48	0	18	0	7
Centroheliozoa	0	0	26	42	0	0
Cercozoa	12	70	51	**543**	**48**	2
Chlorophyta	**1721**	**4527**	63	**707**	0	42
Choanoflagellida	0	0	9	0	26	0
Ciliophora	132	82	**413**	164	0	**112**
Cryptophyta	295	69	132	96	0	22
Diatomea	0	1	11	0	0	0
Dinoflagellata	99	37	180	12	0	0
Discoba	62	21	89	54	2	**92**
Fungi	**401**	78	70	316	0	18
Glaucophyta	12	0	0	0	0	0
Katablepharidophyta	0	0	0	1	0	0
Lobosa	0	2	0	0	0	0
Mesomycetozoa	0	6	0	0	0	0
Metazoa	102	0	40	251	0	49
Ochrophyta	51	**607**	**329**	323	**525**	24
Perkinsea	0	28	**427**	0	11	0
Stramenopiles	114	14	168	**362**	**73**	9
Others	**177**	**201**	278	132	18	**59**
Incertae sedis Eukaryota	0	4	0	0	0	0
Unclassified Eukaryota	164	51	80	321	0	46

M1, module I; M2, module II; M3, module III; M4, module IV; M5, module V; M6, module VI
Boldface indicates the top three highest values
Others, other eukaryotic phyla
Incertae sedis Eukaryota, eukaryotic supergroups with uncertain taxonomic position
Unclassified Eukaryota, eukaryotic groups could not be discriminated at similarity > 80%

Table S6. Numbers of shared OTUs (or nodes) and unique edges (correlations) and their dissimilarity between different microeukaryotic plankton sub-networks based on the three different salinity levels

	Shared OTUs	Shared edges	Unique edges		Dissimilarity of sub-networks
**All**					
Low *vs*. Medium	809	1573	27911	18472	0.9365
Low *vs*. High	300	327	29157	4682	0.9810
Medium vs. High	429	921	19124	4088	0.9265
**Core**					
Low *vs*. Medium	361	527	3982	3656	0.8787
Low *vs*. High	184	174	4335	1430	0.9431
Medium vs. High	216	454	3729	1150	0.8431
**Satellite**					
Low *vs*. Medium	36	11	744	1305	0.9894
Low *vs*. High	7	4	751	578	0.9940
Medium *vs*. High	57	35	1281	547	0.9631

References

Berga M, Zha Y, Szekely AJ, Langenheder S. Functional and compositional stability of bacterial metacommunities in response to salinity changes. Front Microbiol. 2017;8:948.

Breiman, L. Random forests. Machine Learning. 2001;45:5–32.

Campbell BJ, Kirchman DL. Bacterial diversity, community structure and potential growth rates along an estuarine salinity gradient. ISME J. 2013;7:210–20.

Delgado-Baquerizo M, Maestre FT, Reich PB, Jeffries TC, Gaitan JJ, Encinar D. et al. Microbial diversity drives multifunctionality in terrestrial ecosystems. Nat Commun. 2016;7:10541.

Feng S, Powell SM, Wilson R, Bowman JP. Light-stimulated growth of proteorhodopsin-bearing sea-ice psychrophile *Psychroflexus torquis* is salinity dependent. ISME J. 2013;7:2206–13.

Fortmann-Roe S. A3: Accurate, Adaptable and Accessible Error Metrics for Predictive Models. R package version 1.0.0. 2015.

Gao XF, Chen HH, Gu B, Jeppesen E, Xue YY, Yang J. Particulate organic matter as causative factor to eutrophication of subtropical deep freshwater: Role of typhoon (tropical cyclone) in the nutrient cycling. Water Res. 2021;188:116470.

Genuer R, Poggi JM, Tuleau-Malot C. Variable selection using random forests. Pattern Recogn Lett. 2010;31:2225–36.

Green SJ, Blackford C, Bucki P, Jahnke LL, Prufert-Bebout L. A salinity and sulfate manipulation of hypersaline microbial mats reveals stasis in the cyanobacterial community structure. ISME J. 2008;2:457–70.

Herlemann DPR, Labrenz M, Juergens K, Bertilsson S, Waniek JJ, Andersson AF. Transitions in bacterial communities along the 2000 km salinity gradient of the Baltic Sea. ISME J. 2011;5:1571–9.

Ji M, Kong W, Yue L, Wang J, Deng Y, Zhu L. Salinity reduces bacterial diversity, but increases network complexity in Tibetan Plateau lakes. FEMS Microbiol Ecol. 2019;95:fiz190.

Jiao S, Xu YQ, Zhang J, Hao X, Lu YH. Core microbiota in agricultural soils and their potential associations with nutrient cycling. mSystems. 2019;4:e00313–18.

Logares R, Lindstrom ES, Langenheder S, Logue JB, Paterson H, Laybourn-Parry J, et al. Biogeography of bacterial communities exposed to progressive long-term environmental change. ISME J. 2013;7:937–48.

Maestre FT, Quero JL, Gotelli NJ, Escudero A, Ochoa V, Delgado-Baquerizo M. et al. Plant species richness and ecosystem multifunctionality in global drylands. Science. 2012;335:214–8.

Wang J, Wang F, Chu L, Wang H, Zhong Z, Liu Z, et al. High genetic diversity and novelty in eukaryotic plankton assemblages inhabiting saline lakes in the Qaidam Basin. PLoS One. 2014;9:e112812.

Yang J, Ma L, Jiang H, Wu G, Dong H. Salinity shapes microbial diversity and community structure in surface sediments of the Qinghai-Tibetan Lakes. Sci Rep. 2016;6:25078.

Yue L, Kong W, Ji M, Liu J, Morgan-Kiss RM. Community response of microbial primary producers to salinity is primarily driven by nutrients in lakes. Sci Total Environ. 2019;696:134001.

Zhong ZP, Liu Y, Miao LL, Wang F, Chu LM, Wang JL, et al. Prokaryotic community structure driven by salinity and Ionic concentrations in Plateau Lakes of the Tibetan Plateau. Appl Environ Microb. 2016;82:1846–58.

Zou D, Pan J, Liu Z, Zhang C, Liu H, Li M. The distribution of bathyarchaeota in surface sediments of the pearl river estuary along salinity gradient. Front Microbiol. 2020;11:285.
